# Structural basis of HypK regulating N-terminal acetylation by the NatA complex

**DOI:** 10.1038/ncomms15726

**Published:** 2017-06-06

**Authors:** Felix Alexander Weyer, Andrea Gumiero, Karine Lapouge, Gert Bange, Jürgen Kopp, Irmgard Sinning

**Affiliations:** 1 Heidelberg University Biochemistry Center (BZH), INF328, D-69120 Heidelberg, Germany

## Abstract

In eukaryotes, N-terminal acetylation is one of the most common protein modifications involved in a wide range of biological processes. Most N-acetyltransferase complexes (NATs) act co-translationally, with the heterodimeric NatA complex modifying the majority of substrate proteins. Here we show that the Huntingtin yeast two-hybrid protein K (HypK) binds tightly to the NatA complex comprising the auxiliary subunit Naa15 and the catalytic subunit Naa10. The crystal structures of NatA bound to HypK or to a N-terminal deletion variant of HypK were determined without or with a bi-substrate analogue, respectively. The HypK C-terminal region is responsible for high-affinity interaction with the C-terminal part of Naa15. In combination with acetylation assays, the HypK N-terminal region is identified as a negative regulator of the NatA acetylation activity. Our study provides mechanistic insights into the regulation of this pivotal protein modification.

## Abstract (web summary)

N-terminal acetylation is a common eukaryotic protein modification that is primarily catalysed by the N-acetyl transferase complex A (NatA). Here, the authors present the crystal structure of NatA bound to Huntingtin yeast two-hybrid protein K (HypK) and show that HypK is a negative regulator of NatA.

Approximately 85% of all soluble human proteins and more than 60% in yeast carry an acetylation at their N termini^1^,^2^,^3^. Thus, the N-terminal acetylation is one of the most frequent protein modifications in eukaryotes and is implicated in a wide range of biological processes, including cellular apoptosis and growth arrest, protein localization and degradation, enzymatic regulation, ribosome biogenesis and stress response^1^,^4^,^5^,^6^,^7^.

This modification is generally accomplished by N-acetyl transferase complexes (NATs, named NatA to NatG)^4^,^8^,^9^,^10^. The NATs are classified based on their substrate preferences^8^, and most of the complexes consist of at least an auxiliary subunit and a catalytic subunit^11^. Most NATs act during translation but can also bind to ribosomes in the absence of a nascent chain^12^,^13^. Several NAT subunits have been shown to be overexpressed in different types of cancer^14^ and are involved in osteogenesis, neuronal development and human diseases such as Lenz microphthalmia syndrome and Ogden syndrome^5^,^15^,^16^,^17^,^18^,^19^. The NatA complex is responsible for the majority of N-terminal acetylation of proteins with a serine, alanine, threonine, cysteine or valine at their N terminus after the cleavage of the N-terminal methionine by the methionine-amino peptidase^1^,^20^. NatA acts in a co-translational manner and is anchored to the ribosome in close proximity to the exit tunnel^13^,^21^,^22^. It consists of the auxiliary subunit Naa15 and the catalytic subunit Naa10 (refs 20, 21, 22, 23). Structure analysis of the NatA complex from *Schizosaccharomyces pombe* (Sp) revealed an intimate interaction between Naa15 and Naa10. By surrounding Naa10 in a ring-like manner, Naa15 remodels the catalytic site of Naa10 to bring the relevant residues into place for catalysis^20^. While our structural understanding of the NatA complex is rather advanced, only little is known on functional and structural aspects of its regulation.

In higher eukaryotes, Naa50 and Huntington yeast two-hybrid protein K (HypK) have been identified to associate with NatA. The catalytic subunit Naa50 acetylates substrates different from NatA and in complex with NatA forms NatE^1^,^13^,^24^,^25^,^26^,^27^,^28^,^29^. Interestingly, the HypK, which was first identified as an interaction partner of Huntingtin^30^,^31^, is associated with the NatA complex^32^. Moreover, previous data suggested that HypK may play a role in co-translational processes as it co-purifies with NatA and the ribosome-associated complex (RAC)^33^. HypK is specific for higher eukaryotes, plants and several fungi, but is absent in yeast. HypK belongs to the class of intrinsically disordered proteins and shows chaperone activity *in vivo* and *in vitro*^34^,^35^. It interacts with proteins involved in protein folding, cell cycle arrest, response to unfolded proteins, anti-apoptosis and transcription regulation^36^. To better understand the precise role of HypK, we wanted to decipher its crystal structure in the context of NatA and delineate its functional role. Here we present the structural analysis of HypK in complex with NatA from the thermophilic fungus *Chaetomium thermophilum*. Our study shows that HypK acts a negative regulator of the NAT activity of NatA and sets the ground for a molecular understanding of HypK role in co-translational processes.

## Results

### HypK and NatA form a stable complex

**Figure 1:**
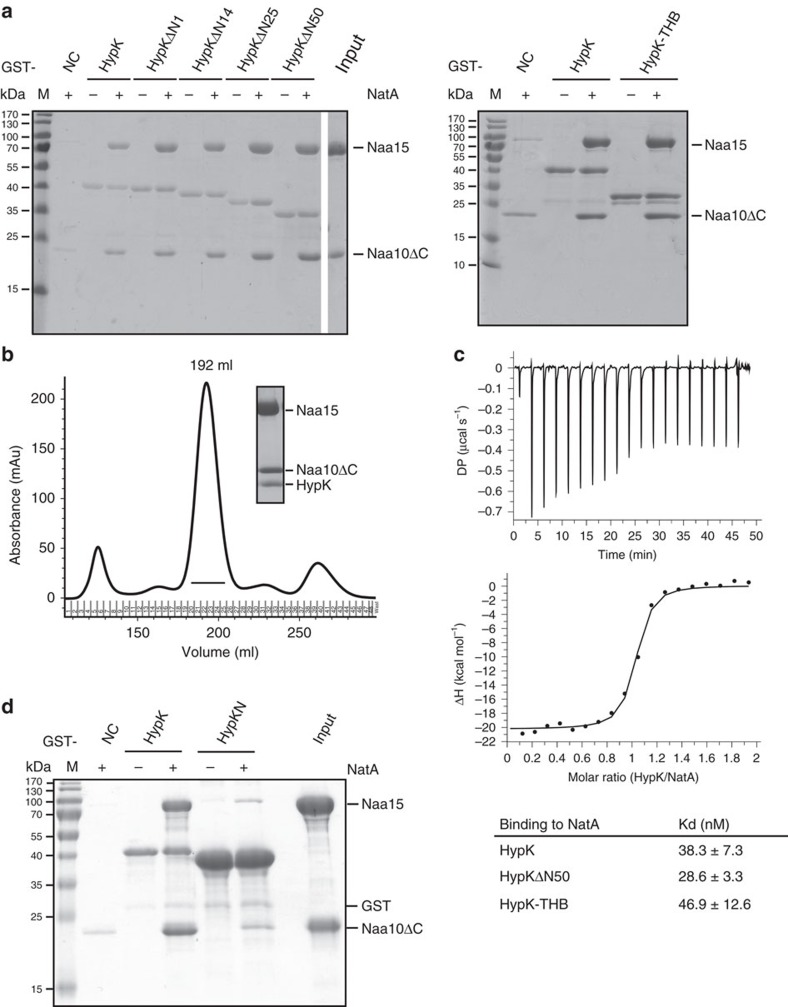
Biochemical characterization of the NatA (Naa15-Naa10ΔC)–HypK complex. (**a**) Pull-down assays probing the binding of the NatA complex to different GST-tagged HypK and variants (HypK, HypKΔN1, HypKΔN14, HypKΔN25, HypKΔN50 or HypK-THB). ‘−' and ‘+' indicate the absence or addition of NatA, respectively. (**b**) SEC elution profile of the NatA–HypK complex. The inset shows a coomassie-stained SDS–PAGE of the peak fraction at 192 ml elution volume. (**c**) Representative isothermal titration calorimetry measurements of complex formation between NatA and HypK (signal is given in differential points, DP). Table of the dissociation constants (*k*_d_) values for relevant ITC measurements. HypK, HypKΔN50 or HypK-THB was injected into the cell containing the preformed NatA complex. The measurements were performed in triplicates. (**d**) GST pull-down assays of the NatA complex with HypK or HypKN (1-82). ‘−' and ‘+' indicate the absence and addition of NatA, respectively. NC, negative control showing no binding of NatA on the beads. Molecular weight markers in the coomassie-stained SDS-PAGEs shown in (**a**) and (**d**) are indicated by ‘M'.

Earlier studies showed that HypK interacts with the NatA complex^32^. To further detail this interaction, we aimed at reconstituting the complex of HypK and NatA, both by pull-down assays using glutathione-S-transferase (GST)-tagged HypK and size exclusion chromatography (SEC; Fig. 1a,b and Supplementary Fig. 1). The conserved orthologous proteins from the thermophilic filamentous ascomycete *C. thermophilum* were used because of their often superior biochemical properties observed in structural studies^37^,^38^ (Supplementary Figs 3–5). *In vitro* pull-down assays and SEC using the full-length Naa15, a variant lacking the 54 C-terminal residues of Naa10 (Naa10ΔC) and full-length HypK, showed the formation of the trimeric complex (Fig. 1a,b). To confirm the stoichiometry of the complex, analytical SEC experiments and multi-angle light scattering (MALS) analysis were carried out and demonstrated the 1:1:1 stoichiometry of the Naa15-Naa10ΔC–HypK complex (Supplementary Fig. 2). To determine the binding affinity of this interaction, isothermal titration calorimetry (ITC) experiments were performed. Titration of the NatA complex with HypK showed the formation of a stable complex with a dissociation constant (*k*_d_) of 38.3±7.3 nM (Fig. 1c, Supplementary Table 1 and Supplementary Fig. 6).

### HypK C terminus is necessary and sufficient for NatA binding

Next, we wanted to delineate the binding region of HypK involved in complex formation with NatA. Secondary structure prediction of HypK using PSIPRED^39^ suggested that HypK contains an unstructured N terminus (residues 1–30), a helical region (residues 31–79) and a three-helix bundle (THB, residues 83–125; Supplementary Fig. 7). On the basis of this prediction, we performed systematic pull-down analysis employing GST-tagged truncation variants of HypK (ΔN1, ΔN14, ΔN25, ΔN50 and -THB (ΔN82)) and the heterodimeric NatA complex. While complex formation was observed with all variants containing the THB, it was abolished when the HypKΔTHB (HypKN, residues 1–82) variant was used (Fig. 1a,d). In addition, ITC measurements confirmed that the full-length HypK, HypKΔN50 and HypK-THB bind with similar affinity to NatA (Fig. 1c, Supplementary Fig. 5 and Supplementary Table 1). Taken together, these results show that the C-terminal region of HypK is necessary and sufficient for high-affinity interaction with NatA.

### Crystal structure of the HypK–NatA complex

**Figure 2:**
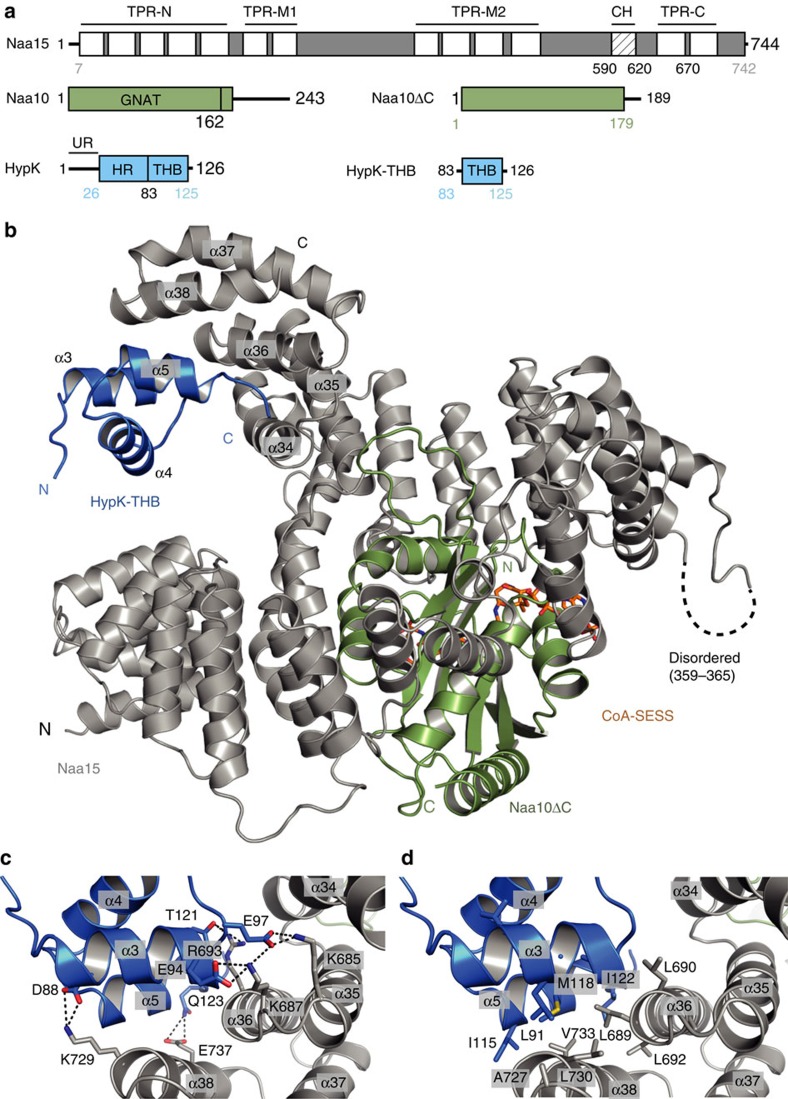
Crystal structure of the NatA–HypK complex. (**a**) Domain architecture of Naa15, Naa10, Naa10ΔC, HypK and HypK-THB. Domains present in the crystal structure are indicated by residue number highlighted in colour (Naa15 in grey, Naa10 in green and HypK in blue). Naa15 comprises 13 TPRs shown as white boxes and clustered in TPR-N, TPR-M1, TPR-M2 and TPR-C. CH, charged helix; GNAT, Gcn5-related acteyltransferase domain; UR, unstructured region; HR, helical region; THB, three-helix bundle. (**b**) The crystal structure of Naa15 (grey) in complex with Naa10ΔC (green), HypK-THB (blue) and the bi-substrate analogue CoA-SESS (orange sticks, residues coloured by element) is shown in ribbon representation. Disordered regions are highlighted with black dashed lines and residue numbers. C- and N termini are indicated. (**c**) Detailed view on the interactions between the Naa15 (grey) and HypK-THB (blue). Salt bridges and hydrogen bonds are highlighted by black dotted lines. (**d**) Close-up view showing the hydrophobic core formed by the interaction between Naa15 and HypK-THB. The hydrophobic residues involved in the interface are shown as sticks.

To characterize the HypK–NatA complex at the molecular level, we determined the structure of the HypK-THB bound to NatA (Naa15-Naa10ΔC). In order to stabilize the complex, a NatA bi-substrate analogue was employed, which comprises Co-enzyme A covalently linked to the N terminus of a Ser-Glu-Ser-Ser peptide (CoA-SESS)^40^ (Supplementary Fig. 8). Crystals of the complex belong to the P 1 2_1_ 1 space group with two heterotrimers in the asymmetric unit. Diffraction data were collected up to 2.6 Å resolution, and the structure was determined by molecular replacement with the *Sp*NatA structure as a search model (PDB: 4KVM^20^) and refined to *R*_work_ and *R*_free_ values of 16.1% and 22.0%, respectively (Table 1). The electron density map was of high quality, showing well-defined density for the CoA-SESS bi-substrate and HypK-THB (Supplementary Fig. 9a). The final model has excellent stereochemistry (Ramachandran allowed 98%). Overall, the structure is well defined, with a low degree of flexibility as indicated by low B-factors (Supplementary Figs 10–12), and only small parts of the structure are disordered (Naa15: residues 1–6, 359–365, 743–744 and the lysine-rich region (charged helix) 573–637; Naa10: residues 180–189; HypK-THB: residue 126), indicated as dotted lines (Fig. 2a,b). The auxiliary subunit Naa15 consists of 38 helices organized in 13 distinct tetratricopeptide repeats (TPRs) adopting a ring-like arrangement. The TPRs can be divided into four major clusters, one at the N terminus (TPR-N, residues 9–178), two central ones (TPR-M1 and -M2, residues 187–254 and 373–519, respectively) and one at the C terminus (TPR-C, residues 638–705), and adopts a ring-like arrangement (Fig. 2a,b and Supplementary Fig. 13a,b). Although TPR-C is predicted to contain two TPRs, the structure reveals that only one TPR is present and that the second TPR is degenerated (Supplementary Fig. 13a,b and Supplementary Table 2). The catalytic subunit Naa10 is located within the centre of Naa15 and establishes an extensive network of interactions with TPR-N, TPR-M1 and TPR-M2 that accounts for a buried surface area of 2,980 Å^2^ (the buried surface area was calculated using PISA^41^). Such an extended contact is important for the stability of the complex and is a conserved feature of N-acetyltransferases^20^,^42^. It comprises a Gcn5-related N-acetyltransferase (GNAT) domain (residues 1–162). An additional helix (α5 residues 162–178) is resolved, which was omitted in the *Sp*Naa10 construct (Fig. 2a,b and Supplementary Fig. 14a,b).

The active site harbours the CoA-SESS bi-substrate analogue, which binds between strands β4 and β5, perpendicular to their plane as described earlier for CoA-SASEA in *Sp*NatA^20^ (Supplementary Fig. 14a). The structure of *Ct*NatA superimposes well with its *Sp*NatA counterpart (with root mean square deviations (r.m.s.d.) of 1.44 and 0.66 Å over 610 and 153 common C_α_ atoms, respectively; Supplementary Fig. 14a). Naa15 interacts with Naa10 via several hydrogen bonds and salt bridges, and is supported by two hydrophobic clusters. Differences are observed in Naa15 TPR-N, and the Naa15 charged helix is disordered in *Ct*Naa15 compared to *Sp*Naa15, which is most likely due to different crystal contacts in both structures (Supplementary Fig. 14c,d). Analysis of the electrostatic surface potential of the HypK–NatA complex showed two positively charged patches, one at the very N terminus of Naa15 and one in a highly charged groove on the opposite site of the complex. This groove is formed by conserved, charged residues in Naa15 and Naa10 (Supplementary Figs 3, 4 and 15). It is noteworthy that the NatA complex binds to the ribosome in a salt-dependent manner^13^, suggesting an ionic interaction between NatA and ribosomal RNA. However, the details of this interaction await further investigation.

### HypK-THB interacts with the Naa15 C-terminal region

During structure building and refinement, extra density appeared in close proximity to Naa15 TPR-C that could be unambiguously assigned to HypK-THB (Fig. 2a,b and Supplementary Fig. 14e). HypK-THB contacts the TPR-C and helix α38 of Naa15 through an extended interface with a buried surface area of 786 Å^2^. The interaction involves hydrogen bonds and salt bridges between HypK (α3, α5, the loop connecting HypK α3 and α4) and Naa15 (α36, α38 and the loop connecting α35 and α36; Fig. 2c and Supplementary Table 3). The contact involves an extended hydrophobic interface between HypK-α3 and α5 (α3: Ala87, Val89, Leu91, Leu92; α5: Ala114, Ile115, Met118, Ile122) and helices α36 and α38 of Naa15 (α36: Leu689, Leu690, Leu692, Leu695; α38: Met726, Ala727, Leu730, Val733, Ile734, Ala736; Fig. 2d). Residues involved in the interaction are well conserved between different HypK orthologues (Supplementary Fig. 5).

### N terminus of HypK binds to the Naa10 active site entrance

**Figure 3:**
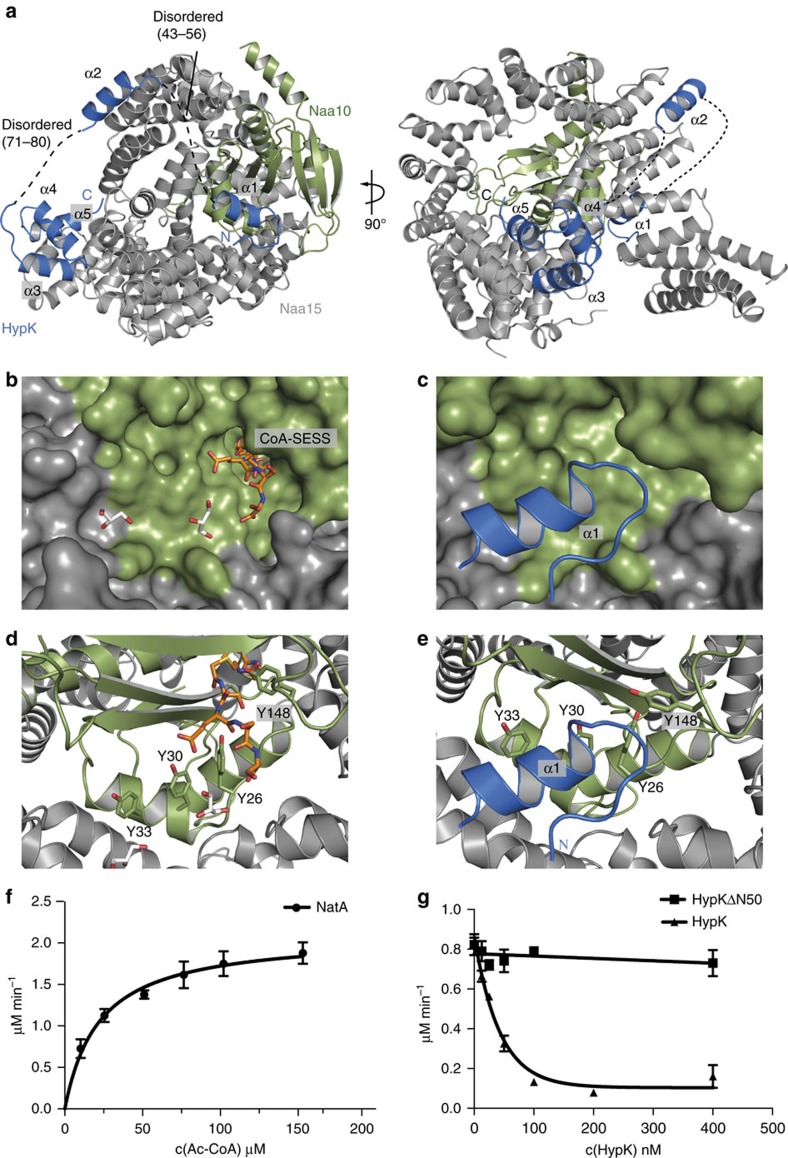
The N terminus of HypK blocks the peptide entry site and modulates NatA activity. (**a**) Overall structure of the Naa15 (grey) in complex with Naa10ΔC (green) and HypK (blue) is shown in ribbon. Disordered regions are indicated with black dashed lines and residue numbers. (**b**) Close-up view on the peptide-binding cleft. NatA is shown as a surface; the substrate analogue is shown as sticks in orange. Two glycerol molecules are represented as white sticks. (**c**) Close-up view on HypK helix α1 binding in proximity to the peptide-binding cleft of Naa10ΔC blocking the entrance for the nascent chain. Naa15 (grey) and Naa10ΔC (green) are shown as a surface; helix α1 of HypK is shown in blue as cartoon. (**d**) Cartoon representation of the HypK-THB–NatA complex. (**e**) Close-up view on the binding side of HypK helix α1 on NatA. (**f**,**g**) Michaelis–Menten curves for (**f**) NatA in the presence of various concentrations of Ac-CoA, c(Ac-CoA), and (**g**) NatA in the presence of different concentrations of HypK (0–400 nM) or HypKΔC50 (0–400 nM) at constant Ac-CoA and SESS-peptide concentrations. A clear decrease in the turn-over number is observed in the presence of HypK. Reactions were performed in triplicates and error bars represent the s.d.

We have shown that the HypK C terminus (THB) is required for the interaction with NatA, while the N terminus is dispensable for high-affinity binding. In order to test the role of the HypK N terminus (HypKN), we determined the crystal structure of full-length HypK bound to NatA (Naa15-Naa10ΔC) at 3.1 Å resolution. Crystals belong to the P 3_2_ space group with two heterotrimers in the asymmetric unit. The structure was solved by molecular replacement using the HypK-THB–NatA complex as a search model and was refined to *R*_work_ and *R*_free_ values of 23.0% and 27.1%, respectively (Table 1). Only small parts of the structure are disordered (Naa15: residues 1–3, 355–364, the lysine-rich region (helix α33) 574–637 and 744; Naa10: residues 176–189; HypK: residues 1–25, 43–56, 71–80 and 126), indicated as dotted lines (Fig. 3a).

This structure shows several surprising features compared to that of the HypK-THB–NatA. First of all, the Naa10 active site is empty, although crystallization was performed in the presence of CoA (Fig. 3b–e), and the Naa15 TPR-N is slightly moved compared to its position in the previous structure (Supplementary Fig. 16). Moreover, two additional pieces of electron density are present on the surface of NatA. These could be attributed to the N terminus of HypK comprising two α helices as predicted by the PSIPRED algorithm^39^ (Supplementary Fig. 7). While one of them locates in close proximity to the Naa10 substrate-binding site, the other one occupies a groove formed by Naa15 helices α8–10 (Fig. 3a). However, the high displacement observed for these regions (B-factor for helix α1 of ∼240 Å^2^ and for helix α2 of ∼230 Å^2^ compared to an average B-factor of 140 Å^2^) and the limited resolution did not allow for the assignment of the corresponding amino-acid sequence. As helix α2 (residues 57–70) contains a single methionine (Met66), we used selenomethionine labelling of HypK to assign helix α2 (Supplementary Fig. 9b,c). Helix α1 was positioned in the remaining density close to the active site, while the connecting loops (residues 43–56 and 71–80) are disordered. HypK helix α1 comprises five hydrophobic residues (Ala36 to Leu40), which bind to a hydrophobic surface and might limit access to the Naa10 active site (Fig. 3b–e and Supplementary Fig. 5).

### N terminus of HypK inhibits the NatA catalytic activity

The position of the HypK N terminus in close proximity of the Naa10 active site suggests that HypK might be involved in modulating the NatA catalytic activity. To test this idea, we first measured the steady-state acetylation rate of NatA in presence of saturating amounts of the tetra-peptide Ser-Glu-Ser-Ser. NatA shows a Michaelis–Menten constant (*K*_m_) of 24±2.4 μM and a *k*_cat_ of 0.35±0.01 s^−1^. Both values are in good agreement with those measured for *Sp*NatA^20^ (Fig. 3f and Table 2). To test the influence of HypK on the catalytic activity of NatA, we measured the steady-state acetylation rate of the NatA complex in presence of increasing amounts of HypK at constant acetyl-CoA and saturating substrate peptide concentrations (Fig. 3g). Using this assay, a significant inhibition of the acetylation activity of NatA by HypK was observed. To determine which part of HypK is responsible for NatA inhibition, we used the HypKΔN50 variant, which lacks helix α1, but still binds NatA with high affinity (Fig. 1a,c). This variant was unable to inhibit the catalytic activity of NatA (Fig. 3g). Taken together, our data show that HypK directly interacts with NatA and efficiently inhibits N-acetylation of a substrate peptide. While HypK-THB is responsible for high-affinity binding to the NatA complex, HypKN is necessary and sufficient for blocking the catalytic activity of the NatA complex.

## Discussion

N-acetylation occurs co-translationally and represents an abundant and universal protein modification. In eukaryotes, it is catalysed by several NATs, of which NatA (Naa15-Naa10) presents the largest range of substrate diversity^3^. In addition, NatA was shown to act in concert with the auxiliary subunits Naa50 and HypK^13^,^24^,^25^,^26^,^32^,^33^. While previous studies indicated the functional importance of HypK^32^,^33^, only little functional and no structural information on this complex was available. HypK is present in higher eukaryotes including human and plants and most fungal species, with bakers yeast as the exception^32^,^43^. Our study identifies a HypK homologue in the thermophilic fungus *C. thermophilum* and shows that HypK interacts with NatA, forming a stable heterotrimeric complex. Previously, HypK has been characterized as an intrinsically unstructured protein^34^,^35^, and we noted that a carrier protein, such as GST, significantly improves its behaviour during purification. Our structural analyses show, however, that in the context of NatA the C-terminal region of HypK forms a THB responsible for high-affinity binding to the C terminus of Naa15. The interface is formed by an elaborate network of hydrophobic and electrostatic interactions. The high nano-molar affinity between HypK and NatA suggests that HypK might be an intrinsic partner of the NatA complex. Interestingly, the HypK-THB shows a high degree of conservation between different HypK homologues^43^, suggesting that this interaction might be generally used as the interaction site within the HypK–NatA complex. Our study also shows that the regulatory function of HypK resides in its N-terminal region comprising the first 50 residues (disordered N terminus and helix α1), which bind at the entrance of the Naa10 active site and seem to block access to the active site. Regulation of NatA function by HypK has been previously reported^32^. However, in that study a reduction in acetylation of the known NatA substrate PCNP was observed in HypK knockdown HeLA cells. While this discrepancy cannot be resolved at present, it might be due to differences in the experimental set-up, such as different organisms (fungus or human) were employed and an *in vitro* reconstituted complex was used compared to components derived from co-immunoprecipitation.

Nα-acetylation occurs predominantly co-translationally in eukaryotes^12^,^13^,^21^,^44^. In particular, the NatA complex was shown to bind the ribosome in yeast^13^, human^21^,^22^ and recently in *Arabidopsis thaliana*^7^, and a non-ribosome-bound, cytosolic fraction of NatA was also identified in human and plants^7^,^21^,^22^. In addition, early crosslinking data suggested that the NatA complex interacts with the ribosomal proteins uL29 and uL23 (ref. 12), which in yeast also bind the methionine aminopeptidase Map1 (ref. 45). Cleavage of the N-terminal methionine by Map1 occurs before NatA acetylates the nascent chain, suggesting that Map1 and NatA have to act on the nascent chain in a highly coordinated and sequential manner. In this study, we identified positively charged patches in NatA, which might be involved in interaction with ribosomal RNA. Similar interactions between positively charged regions and ribosomal RNA have been reported for a number of ribosome-associated factors, including the chaperone Ssb^46^ and RAC^47^, as well as for the human signal recognition particle SRP^48^.

**Figure 4:**
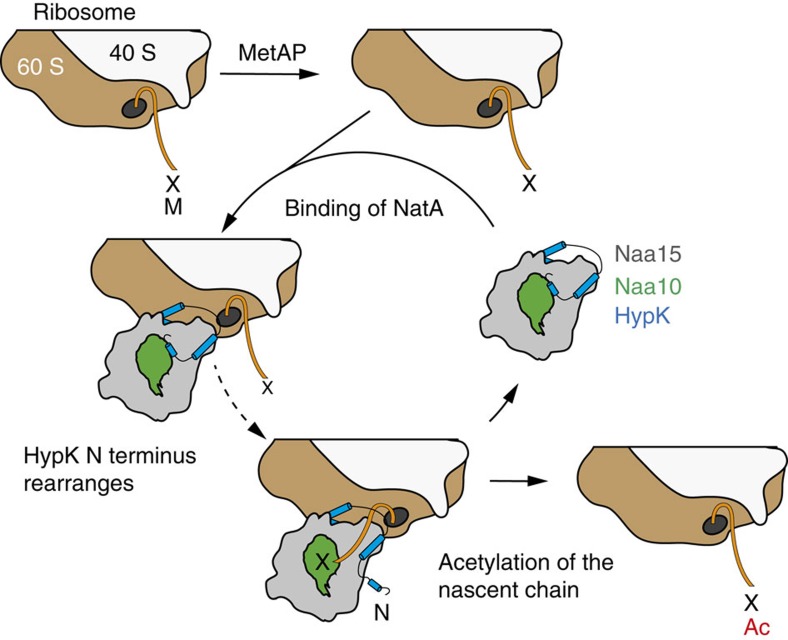
Model describing the interaction between HypK and NatA. HypK (blue) binds onto NatA (Naa15 in grey and Naa10 in green) via the C-terminal THB and blocks the peptide entrance of Naa10 via the N-terminal helix α1. The HypK–NatA complex binds to the ribosome exposing a substrate nascent chain (orange) after the cleavage of the initial methionine by a methionine aminopeptidase (MetAP). After binding, helix α1 at the N terminus rearranges, the nascent chain gets acetylated and NatA gets released from the ribosome. X=for example, serine, alanine, glycine, valine or cysteine. M, methionine. The ribosomal subunits 60 S (wheat) and 40 S (white) are indicated. HypK N terminus is indicated. Ac, acetyl.

We propose the following model for the role of HypK in Nα-terminal acetylation. HypK binds to the C terminus of Naa15 via its THB, while HypK N terminus containing helix α1 binds in proximity of Naa10 peptide entry site and blocks the interaction with the substrate nascent chain (Fig. 4). This interaction might already take place in the cytosol before the binding of NatA to the ribosome. As HypK was found at ribosomes^32^,^33^ and because of the high affinity of HypK for NatA, we suggest that HypK is permanently bound to NatA. As the ribosomal nascent chain gets acetylated by NatA, the N-terminal helix α1 of HypK has to re-localize. As the N terminus is dispensable for high-affinity binding of HypK, such a rearrangement seems feasible. Re-localization could be triggered by the emerging nascent chain, by an interaction of helix α1 with the ribosome or by binding of an additional ribosome-associated factor. One plausible candidate for such interaction could be the RAC residing also in close proximity to the exit tunnel^49^,^50^. Interestingly, a heterotrimeric NatA–RAC complex was already suggested to form in mammals^33^. However, further work is necessary to validate this hypothesis. Numerous co-translational factors including targeting factors, enzymes and chaperones compete for access to the nascent chain. We are still in the process of dissecting them one-by-one in order to finally arrive at a comprehensive understanding of their interplay at the ribosomal tunnel exit.

## Methods

### Construction of expression plasmids

Naa15 and Naa10 coding sequences were PCR-amplified using *C. thermophilum* cDNA as template and a set of primers (Supplementary Table 4). The PCR product was ligated into the TOPO Vector (TOPO TA Cloning Kit, Invitrogen). The NcoI restriction sites present in the coding sequences were mutated by site-directed mutagenesis (QuikChange Lightning, Agilent Technologies). The *NAA15* gene was PCR-re-amplified , digested and ligated directly into the pET24d vector (Novagen) or into a modified pET24d vector containing an N-terminal His_6_-TEV (tobacco-etch-virus) site (G. Stier, Heidelberg)-digested NcoI/BamHI resulting into Naa15, His_6_-TEV-Naa15 or His_6_-Naa15. The full-length *NAA10* gene was PCR-amplified, digested and ligated into pET24d vector-digested NcoI/BamHI (Naa10). The *NAA10* gene lacking the last 54 residues were re-amplified by PCR and fused to a C-terminal His_6_, digested and ligated into the pET24d vector restriction sites NcoI/BamHI (Naa10-His_6_). The *HYPK* gene fused to an N-terminal His_6_ tag was cloned into the NcoI and BamHI restriction sites of the pET24d vector (His_6_-HypK). The *HYPK* gene was also cloned into pETGST1a vector (G. Stier) containing a GST tag followed by a TEV protease recognition sequence (GST-HypK). The truncated GST-HypK variants (ΔN1 (2–126), ΔN14 (15–126), ΔN25 (26–126), ΔN50 (51–126), THB (83–126) and ΔC44 (1–82)) were constructed by site-directed mutagenesis. A C-terminal STREPII-tag was fused to the GST-HypK, GST-HypKΔN50 or GST-HypK-THB by site-directed mutagenesis (QuikChange Lightning, Agilent Technologies). All constructs where verified by sequencing (MWG Biotech, München). Our Naa15 variant presented two mutations compared to the published *C. thermophilum* sequence, N707K and G724D. However, the same mutations are found in other thermophilic homologues (Supplementary Fig. 3).

### Expression and purification of native proteins

All native proteins were expressed in Rosetta II (*DE3*) *E. coli* strain (Novagen). Cells were grown at 30 °C for 14 h in LB-Media supplemented with lactose (17.5 g l^−1^), kanamycin (50 μg ml^−1^) and chloramphenicol (34 μg ml^−1^). The GST-HypK variants were purified as follows: cells were harvested, resuspended in lysis buffer (20 mM HEPES pH 8.0, 250 mM NaCl, 20 mM MgCl_2_, 20 mM KCl, 40 mM Imidazole) and lysed using a Microfluidizer (M1-10L, Microfluidics). After centrifugation at 4 °C for 20 min at 64,000*g*, the cleared lysate was loaded on a 2 ml Histrap column (IMAC (immobilized metal affinity chromatography) purification, GE Heathcare), washed with lysis buffer (20 column volumes) and eluted with the lysis buffer containing 300 mM imidazole. For the GST-HypK-STREPII-tag variants an additional step was added to cleave the GST carrier. After the Histrap column, TEV cleavage was performed during dialysis overnight at 4 °C in dialysis buffer (20 mM HEPES pH 8.0, 150 mM NaCl, 20 mM MgCl_2_, 20 mM KCl and 1 mM Tris-(2-carboxyethyl)-phosphin). The carrier was removed by reverse IMAC. All the proteins were then further purified by SEC using a S75 gel-filtration column (GE Healthcare) in the SEC buffer (20 mM HEPES pH 7.5, 150 mM NaCl, 10 mM MgCl_2_ and 10 mM KCl). For the preparation of the Naa15–Naa10ΔC-His_6_ dimeric complex, individual cell lysates were mixed and IMAC-purified. For the His_6_-TEV-Naa15–Naa10 dimeric complex used for gel mobility shift assays, a TEV cleavage step was performed during dialysis overnight at 4 °C in dialysis buffer (20 mM HEPES pH 8.0, 150 mM NaCl, 20 mM MgCl_2_, 20 mM KCl and 1 mM Tris-(2-carboxyethyl)-phosphin). The His_6_ tag was removed by reverse IMAC. The eluate was then diluted 10 × with SP-loading buffer (50 mM citrate pH 5.5, 100 mM NaCl), loaded on a 5 ml SP-Sepharose ion exchange column (GE Healthcare) and eluted with a salt gradient ranging from 100 to 1,100 mM NaCl in SP-loading buffer. The dimer was then loaded onto a S200 gel-filtration column (GE Healthcare) in the SEC buffer. For the preparation of the His_6_-Naa15-Naa10ΔC-His_6_-His_6_-HypK trimeric complex, individual cell lysates were mixed, IMAC-purified and loaded onto a S200 gel-filtration column (GE Healthcare) in the SEC buffer.

### Selenomethionine-labelled HypK expression and purification

His_6_-HypK was expressed in *E. coli* BL21(DE3; Novagen). Cells were grown in M9 medium supplemented with 125 mg l^−1^ lysine, 125 mg l^−1^ threonine, 125 mg l^−1^ phenylalanine, 50 mg l^−1^ valine, 50 mg l^−1^ leucine, 50 mg l^−1^ isoleucine, 5 g l^−1^ glucose, 250 mM MgCl_2_, 1 mM CaCl_2_ and 50 mg l^−1^ seleno-L-methionine. Protein expression was induced at an OD_600_ of 0.6 by the addition of 1 mM isopropyl-β-D-thiogalactosides. After 3–6 h of growth, cells were harvested. Bacterial pellets of His_6_-Naa15-Naa10ΔC-His_6_ were co-lysed with the selenomethionine-labelled HypK. The purification was performed as described for the native complex.

### Crystallization and data collection

The NatA–HypK-THB complex (Naa15, Naa10ΔC-His_6_ and HypK-THB-StrepII) was formed by titrating the Naa15, Naa10ΔC-His_6_ dimeric complex with HypK-THB-StrepII. The trimeric complex (15 mg ml^−1^) was mixed with 1 mM bi-substrate analogue CoA-SESS (ref. 40: CoA-Ac-SES4; Peptides Specialty Laboratories GmbH, Heidelberg) and incubated overnight at 4 °C. The complex was mixed 1:1 with crystallization buffer, and crystals appeared after 5 days in 0.1 M Tris pH 8.5 and 25% PEG3350.

The NatA–HypK complex (His_6_-Naa15, Naa10ΔC-His_6_ and full-length His_6_-HypK) crystallized at a concentration of 30–45 mg ml^−1^ in the presence of 1 mM CoA. Crystals appeared after 3 days at 291 K in 0.2 M Mg(NO_3_)_2_ and 20% PEG3350. Crystallization for the selenomethionine-labelled HypK in complex with NatA (His_6_-Naa15, Naa10ΔC-His; 25–30 mg ml^−1^) was performed at 291 K by the hanging-drop vapour-diffusion method using glass cover slides. Crystals appeared after 3 days in 0.2 M Mg(NO_3_)_2_ and 20% PEG3350. All crystals were cryoprotected by soaking in 20% glycerol containing mother liquor and flash-frozen in liquid nitrogen.

Data sets were collected at BM14 and ID23-2 at the European Synchrotron Radiation Facility (ESRF) at cryogenic temperature. The data set of HypK–NatA was collected on the selenomethionine-labelled protein at the selenium peak wavelength. The data were processed using XDS^51^ and scaled with aimless^52^ within the CCP4 package^53^. The structure of HypK–THB–NatA was solved by molecular replacement using the *S. pombe* NatA complex (4KVM (ref. 20)) as a search model with phaser-MR^54^ within the Phenix package^55^. The structure of the HypK–NatA complex was solved by molecular replacement using the HypK-THB–NatA structure as model. Model building and refinement were performed using Phenix.refine^56^, Refmac5 (ref. 57) and MolProbity^58^ within the Phenix programme suite^56^ and *Coot*^59^. Figures were prepared in PyMOL (Molecular Graphics System, Version 1.8 (ref. 60; http://www.pymol.org)). Electrostatic surface potentials were calculated with APBS and PDB2PQR^61^,^62^ integrated in PyMOL (Molecular Graphics System, Version 1.8 (ref. 60; http://www.pymol.org)). Sequence alignments were performed using Clustal Omega^63^ and visualized with ESPript 3.0 (ref. 64; http://www.espript.ibcp.fr).

### Pull-down assays

GST beads (20 μl slurry, GE Healthcare) were loaded on spin columns and equilibrated with PBS (10 mM Na_2_HPO_4,_ 1.8 mM KH_2_PO_4_, pH 7.4, 137 mM NaCl, 2.7 mM KCl). The beads were loaded with a saturating amount of purified GST-HypK variants, incubated for 10 min on a rotating wheel at 4 °C and centrifuged for 2 min at 4,000*g*. A threefold molar excess of the dimeric NatA complex (Naa15-Naa10ΔC-His_6_) was then added to the beads and incubated for 10 min on a rotating wheel at 4 °C. The columns were washed with 3 × 400 μl PBS and centrifuged for 2 min at 4,000*g*. The proteins were eluted with 40 μl of GST-elution buffer (50 mM Tris/HCl pH 8.0, 10 mM reduced glutathione), and the protein qualitative assessment performed on 15% SDS–PAGE and using coomassie staining. The uncropped coomassie SDS–PAGE gel is shown in Supplementary Fig. 17.

### Acetyltransferase assay and inhibition assay with HypK

NatA acetyltransferase activity was determined using a 96-well plate reader spectrophotometer (SpectraMax M5e Multi Mode Microplate reader, Molecular Devices) with a method adapted from ref. 65. Acetylation reaction, monitored at 412 nm and performed at 30 °C, was started by the addition of 50 μl of the NatA complex (200 nM, Naa15-Naa10ΔC-His_6_) to 50 μl of pre-incubated reaction solution containing 50 mM HEPES pH 7.4, 2 mM EDTA, 1 mM 5,5′-dithio-bis-2-nitrobenzoic acid, 1.5 mM SESS-peptide (PSL Peptide Specialty Laboratories GmbH, Heidelberg) and various concentrations of acetyl-CoA. Control reactions were performed in the absence of the NatA complex and in the absence of the substrate SESS-peptide where no activity was detected in these conditions. For the inhibition assay, the NatA complex (100 nM) was incubated with different amounts of full-length HypK-StrepII or HypKΔN50-StrepII (ranging from 0 to 400 nM) at 30 °C for 10 min prior to addition to the reaction solution. Activity was measured at constant concentration of CoA.

### ITC measurements

Binding experiments were performed using a MicroCal VP-ITC and a PEAQ-ITC microcalorimeter (MicroCal and Malvern Instrument GmbH, respectively) equilibrated at 25 °C. Protein samples were dialysed overnight at 4 °C against ITC buffer (20 mM HEPES pH 7.5, 150 mM NaCl, 10 mM MgCl_2_ and 10 mM KCl) and the samples were degassed immediately before the measurement. Protein concentrations ranging between 10 and 40 μM in the cell (NatA complex: Naa15-Naa10ΔC-His_6_) and 100 and 400 μM in the syringe (HypK-Strep II variants) were used in the experiment. The heats of dilution were subtracted from the raw titration before data analysis. The data were fitted using a single-site binding model and analysed using either Origin (V. 7.0) or the MicroCal PEAQ-ITC analysis software.

### Analytical SEC

For analytical SEC experiments, purified components (NatA, NatA-HypK or HypK) were run on a Superdex 200 10/300 gel-filtration column (GE Healthcare) equilibrated in SEC buffer using a ÄKTA pure chromatography system (GE Healthcare). The peak fraction was analysed by coomassie-stained SDS–PAGE. The uncropped coomassie SDS–PAGE gel is shown in Supplementary Fig. 18.

### Multi-angle light scattering

The NatA-HypK complex (0.2 mg) was injected onto a Superdex 200 10/300 gel-filtration column (GE Healthcare) in SEC buffer coupled to a MALS system (Daw Heleos II 8+ and Optilab T-rEX, Wyatt Technology). Data were analysed using the Astra 6 software (Wyatt Technology).

### Data availability

Coordinates and structure factors have been deposited with the Protein Data Bank under the accession codes 5NNP and 5NNR. The data that support the findings of this study are available from the corresponding author upon reasonable request.

## Additional information

**How to cite this article:** Weyer, F. A. *et al*. Structural basis of HypK regulating N-terminal acetylation by the NatA complex. *Nat. Commun.* **8,** 15726 doi: 10.1038/ncomms15726 (2017).

**Publisher's note:** Springer Nature remains neutral with regard to jurisdictional claims in published maps and institutional affiliations.

## Supplementary Material

Supplementary Information Supplementary figures, supplementary tables and supplementary references.

## Figures and Tables

**Table 1:** Data collection and refinement statistics (molecular replacement–single anomalous dispersion).

	**Naa15-Naa10**Δ**C-HypK-THB**	**Naa15-Naa10**Δ**C-HypK (Se-Met)**
*Data collection*		
Space group	P 1 2_1_ 1	P 3_2_
Cell dimensions		
*a*, *b*, *c* (Å)	93.3, 106.0, 130.9	85.4, 85.4, 319.6
α, β, γ (°)	90.0, 95.0, 90.0	90.0, 90.0, 120.0
Resolution (Å)	49.3–2.6 (2.69–2.6)*	48.4–3.1 (3.21–3.1)*
*R*_merge_	16.0 (95.9)	14.2 (216.2)
*I*/σ*I*	8.9 (1.7)	13.8 (1.2)
Completeness (%)	99.7 (97.3)	100.0 (100.0)
Redundancy	6.9 (6.5)	11.7 (11.8)
		
*Refinement*		
Resolution (Å)	49.3–2.6 (2.69–2.6)*	42.3–3.1 (3.2–3.1)
No. of reflections	77,891 (7,551)	47,259 (4,745)
*R*_work_/*R*_free_	16.1/22.0	23.0/27.1
No. of atoms		
Protein	14,318	14,720
Ligand/ion	340	0
Water	443	0
*B*-factors		
Protein	55.90	140.7
Ligand/ion	73.00	
Water	46.70	
R.m.s. deviations		
Bond lengths (Å)	0.007	0.009
Bond angles (°)	0.97	1.45

Each structure was determined from one crystal.

^*^Values in parentheses are for highest-resolution shell.

**Table 2:** Steady-state kinetics for *Ct*NatA and *Sp*NatA*.

	***Ct*****NatA**	***Sp*****NatA**
*k*_cat_ (s^−1^)	0.35±0.01	3.0±0.5
*K*_m_ (μM)	24±2.4	59±5
Experimental temperature (°C)	30	25

^*^^20^.
