# Supplementary material for: Structural basis of HypK regulating N-terminal acetylation by the NatA complex
Source: Nat Commun. 2017 Jun 6;8:15726. doi: 10.1038/ncomms15726 (PMC5467210; doi:10.1038/ncomms15726)
Supplement: Supplementary Information — Supplementary figures, supplementary tables and supplementary references. [file ncomms15726-s1.pdf]

Supplementary Table 1

ITC-analysis of the NatA (Naa15-Naa10ΔC) complex interaction with different HypK variants.

	**HypK**	**HypKΔN50**	**HypK-THB**
**K_d (nM)**	38.3 ± 7.3	28.6 ± 3.3	46.9 ± 12.6
**n**	0.98 ± 0.006	0.93 ± 0.03	1.13 ± 0.009
**ΔH (kcal mol^{-1})**	- 20.3 ± 0.2	- 9.29 ± 0.05	- 10.1 ± 0.01
**-TΔS (kcal mol^{-1})**	10.2	- 0.92	52.4
**ΔG (kcal mol^{-1})**	- 10.1	- 10.2	42.3

Supplementary Table 2

Exact sequence of the TPRs in *Ct*Naa15 as predicted by the TPR-prediction server[1] (https://toolkit.tuebingen.mpg.de/tprpred)**.**

**Cluster**	**TPR**	**Start residue**	**End residue**	**Sequence**
TPR-N	1	9	42	EANLFRTVIRHYEDKQYKRGLKAAEQILKKNPKH
TPR-N	2	43	76	GDTMSMKALILNAQGKTEEAFALAKEALTIDMKS
TPR-N	3	77	110	YICWHVYGILYRTNKNFDEAIKAYKFALKLEPES
TPR-N	4	111	144	HQIQRDLAVLQIQMRDYAGYVQSRLNMLKARPQI
TPR-N	5	145	178	RQNWTALAIAYHLEGNLEKAEHILTTYEKSLTTP
TPR-M1	6	187	220	SEALLYKNTIIAERGDIERALQHLETDCKHCLDR
TPR-M1	7	221	254	LAVMELRASYLSKLARKDEAAKAYRALLDRNPEH
TPR-M2	8	373	406	ALYYLAQHYNYYMSRDLTRALEYVEKAIELDPKN
TPR-M2	9	407	440	VDFHMTKARIFKHQGDLAKAAETMDYARSLDPKD
TPR-M2	10	443	476	INSKAAKYQLRNNENEKALATMGLFTRAETAGGP
TPR-M2	11	486	519	IWFLTEDGEAWQRRGNTALALKRYHTVFSIFDTW
TPR-C	12	638	671	KDDDPNGEKLAATKDPLGDAMKFLNYILQFSPKN
TPR-C	13	672	705	IDGQIAGFEVYIRKKKYLLALRCLKAASAIDKNH

Supplementary Table 3

Interactions between HypK-THB and Naa15 as evaluated using PISA from CCP4[2].

	**HypK-THB**	**Naa15**
**Hydrogen bonds**	α3: Asp88	α38: Lys729
	α3: Glu94	loop α35-α36: Lys687
	α5: Gln123	α38: Glu737
	loop α3-α4: Glu97	loop α35-α36: Lys685
	α5: Tyr121	α36: Arg693
	α5: Gln123	α36: Arg696
**Salt bridges**	α3: Asp88	α38: Lys729
	α3: Glu94	loop α35-α36: Lys687
	loop α3-α4: Glu97	loop α35-α36: Lys685
	loop α3-α4: Glu97	loop α35-α36: Lys687

Supplementary Table 4

Primers used in this study.

Name of primer	Sequence 5' 3'
ct-Nat1-F	ATGCCGCAGCCACTGAG
ct-Nat1-R	CTAGGCGCCAGGCACCA
Nat-dNco-A-F	GGCTGCAGAAACTATGGACTATGCGCGG
Nat-dNco-A-R	CCGCGCATAGTCCATAGTTTCTGCAGCC
Nat-dNco-B-F	GCCCTTGCCACTATGGGCTTGTTCACTC
Nat-dNco-B-R	GAGTGAACAAGCCCATAGTGGCAAGGGC
Nat1-NcoI-oH-F	TTAACCATGGGCCCGCAGCCACTGAGTAC
Nat-Nco-H6-F	TTAACCATGGGCCACCATCACCATCACCATCCGCAGCCACT GAGTAC
Nat1-Bam-R	TTAAGGATCCTTAGGCGCCAGGCACACCAAC
Ard1-delNco-F	GAGCCAACTAGCAATGGTAGAAACCTAC
Ard1-delNco-R	GTAGGTTTCTACCATTGCTAGTTGGCTC
Ard1-Nco-F	TTAACCATGGACATCCGCCTCCTCCG
Ard1-6H-Bam-R	TTAAGGATCCTTAATGGTGATGGTGATGGTGTGTATGCTTA CTCTCATCC
Ard1-Bam-R	TTAAGGATCCTTATGTATGCTTACTCTCATCCC
Ard1-dC54-R	TTAAGGATCCTTAATGGTGATGGTGATGGTGATTAACGCCA TTCGAACCTGCAGC
HypK-NcoI-6H-F	TTAACCATGGGCCACCATCACCATCACCATGCTG CCGAGGACAGACAGCCC
HypK_Nco_F	TTAACCATGGGCGCTGCCGAGGACAGACAGCCC
HypK-BamHI-R	TTAAGGATCCTTAAAAAGCAGGCTGGATATAAGCCTTCA
ctHypKDN1CStrep_F	GTCTGTCCTCGGCAGCCTGAAAATAAAGATTCTCAG
ctHypKDN1CStrep_R	CTGAGAATCTTTATTTTCAGGCTGCCGAGGACAGAC
ctHypKDN14CStrep_F	CTGAGAATCTTTATTTTCAGGCCACCGCAGGCGATG
ctHypKDN14CStrep_R	CATCGCCTGCGGTGGCCTGAAAATAAAGATTCTCAG
ctHypKDN25CStrep_F	GAGAATCTTTATTTTCAGGCCCCGGCCGCCAAG
ctHypKDN25CStrep_R	CTTGGCGGCCGGGGCCTGAAAATAAAGATTCTC
HypKDC44_Ba_R	AATTAAGGATCCTTACTTCTTGACCTCCTTCTTCTCC
HypKdN50_Nco_F	TTAATTCCATGGCCCCGGCCCGCGAAG
HypK_THB_Nco_F	TTAATTCCATGGGGAAGGTCGACCCGGCTGATGT
ctHypK_CStrepII_R	TTAATTAAGCTTTTATTTTTCGAACTGCGGGTGGCTCCAGC TACCACTACCGGATCCAAAAGCAGGCTGGATATAAGCCTT

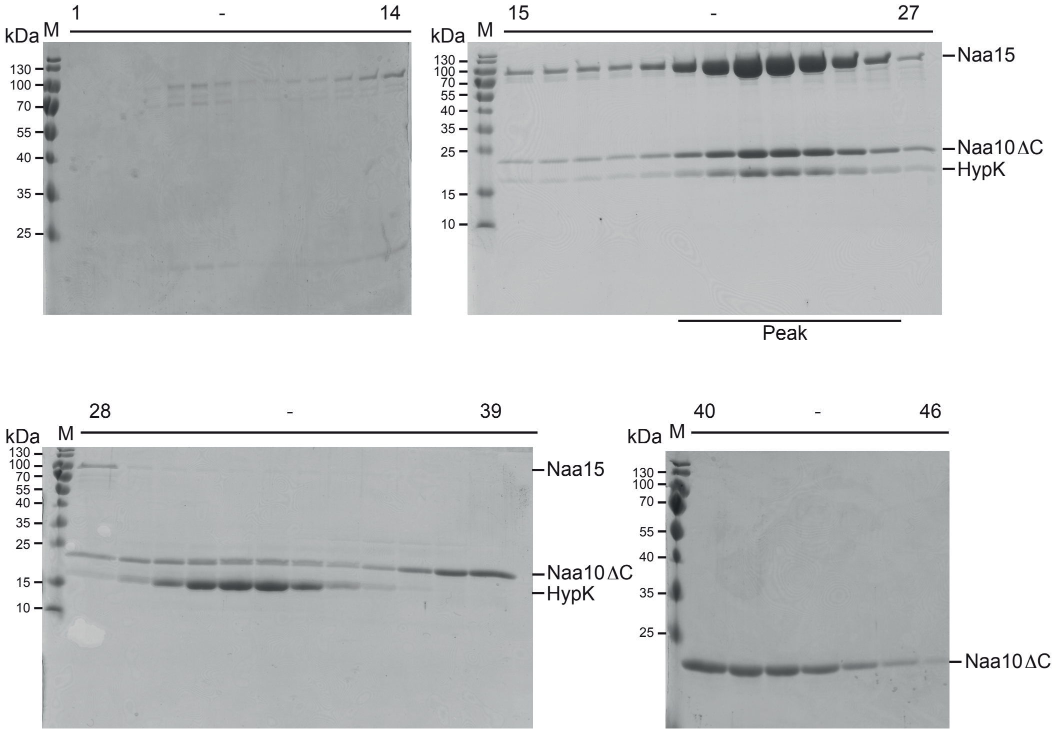

Supplementary Figure 1: Purification of the HypK - NatA complex. Uncropped SDS-PAGE showing all SEC fractions of **Fig. 1b**. M: protein ladder. Peak: fractions from the SEC peak (**Fig. 1b**).

a

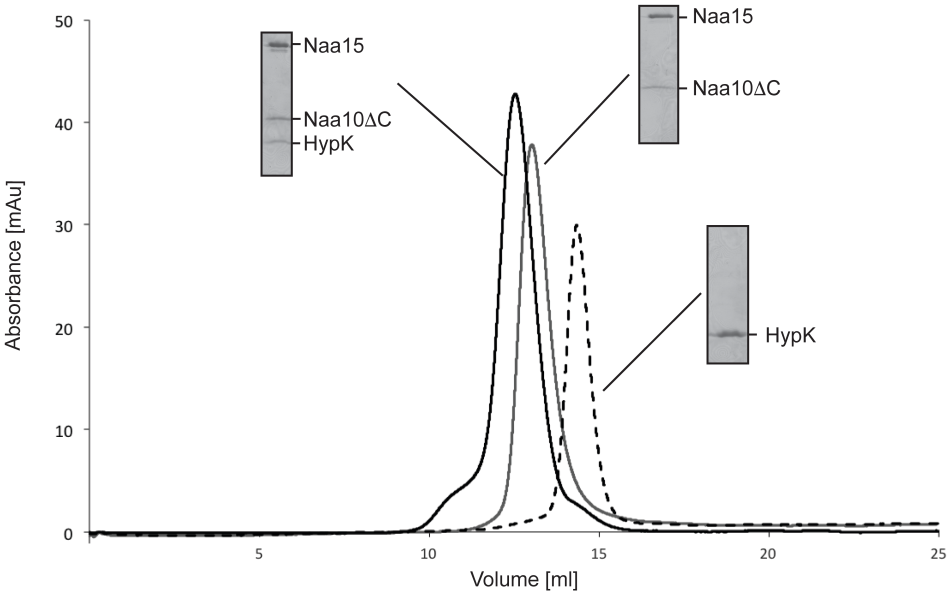

b

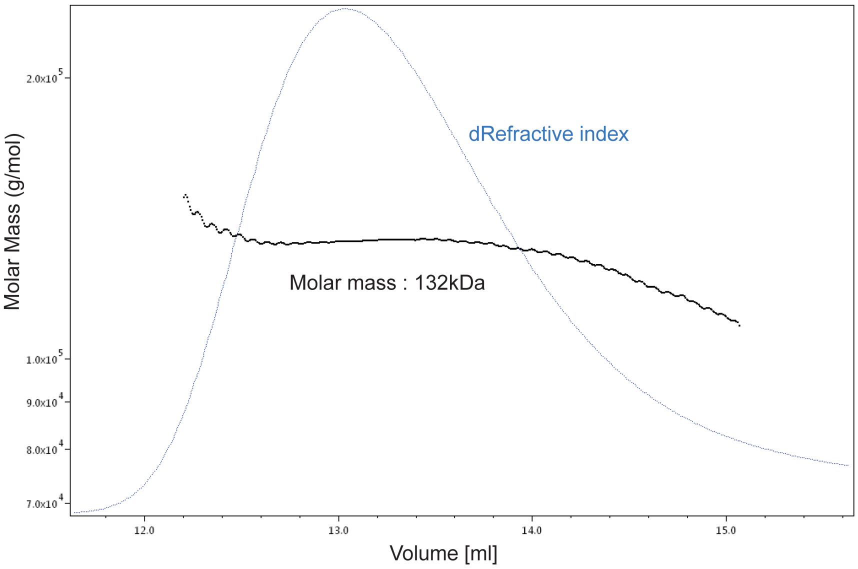

Supplementary Figure 2: Analytical size exclusion chromatography and SEC-MALS of the NatA - HypK complex. (a) Sequential runs of NatA (Naa15-NaaΔC, gray line), HypK (black dotted line) and NatA-HypK (black line) on a Superdex S200 10/300. The peak was analyzed by SDS-PAGE (shown as an inset). (b) SEC-MALS analysis of the NatA - HypK complex (theoretical calculated molar mass: 122 kDa). The differential refractive index is shown as blue line, the absolute molecular mass as black dotted line.

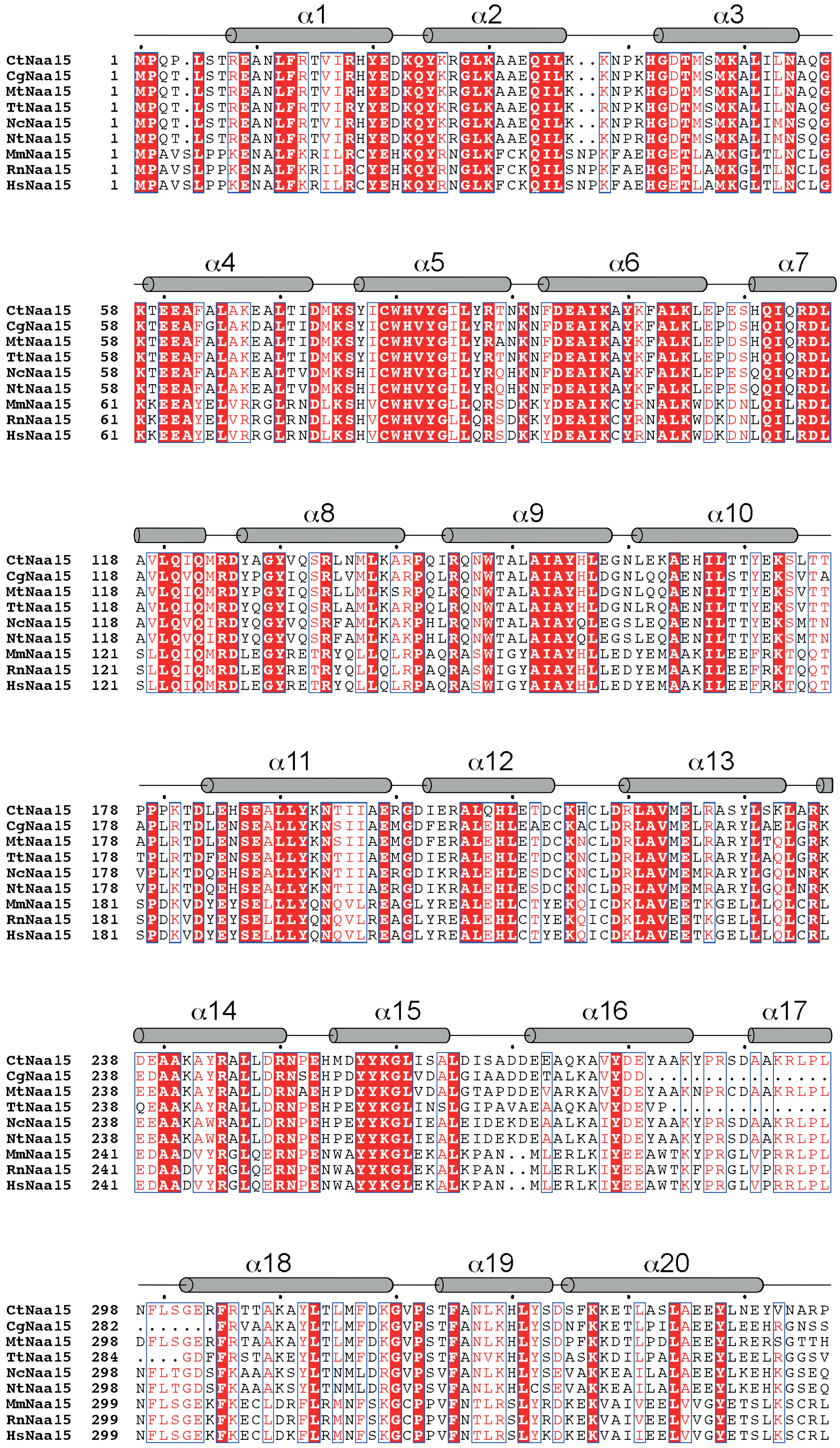
α1 α2 α3
CtNaa15 1 MPQP.LSTREANLFRTVIRHYEDKQYKRGLKAAEQILK..KNPKHGDTMSMKALILNAQG
CgNaa15 1 MPQT.LSTREANLFRTVIRHYEDKQYKRGLKAAEQILK..KNPKHGDTMSMKALILNAQG
MtNaa15 1 MPQT.LSTREANLFRTVIRHYEDKQYKRGLKAAEQILK..KNPKHGDTMSMKALILNAQG
TtNaa15 1 MPQT.LSTREANLFRTVIRYYEDKQYKRGLKAAEQILK..RNPKHGDTMSMKALILNAQG
NcNaa15 1 MPQT.LSTREANLFRTVIRHYEDKQYKRGLKAAEQILK..KNPRHGDTMSMKALIMNSQG
NtNaa15 1 MPQT.LSTREANLFRTVIRHYEDKQYKRGLKAAEQILK..KNPRHGDTMSMKALIMNSQG
MmNaa15 1 MPAVSLPPKENALFKRILRCYEHKQYRNGLKFCKQILSNPKFAEHGETLAMKGLTLNCLG
RnNaa15 1 MPAVSLPPKENALFKRILRCYEHKQYRNGLKFCKQILSNPKFAEHGETLAMKGLTLNCLG
HsNaa15 1 MPAVSLPPKENALFKRILRCYEHKQYRNGLKFCKQILSNPKFAEHGETLAMKGLTLNCLG
α4 α5 α6 α7
CtNaa15 58 KTEEAFALAKEALTIDMKSYICWHVYGILYRTNKNFDEAIKAYKFALKLEPESHQIQRDL
CgNaa15 58 KTEEAFGLAKDALTIDMKSYICWHVYGILYRTNKNFDEAIKAYKFALKLEPDSHQIQRDL
MtNaa15 58 KTEEAFALAKEALTIDMKSYICWHVYGILYRANKNFDEAIKAYKFALKLEPDSHQIQRDL
TtNaa15 58 KTEEAFALAKEALTIDMKSYICWHVYGILYRTNKNFDEAIKAYKFALKLEPDSHQIQRDL
NcNaa15 58 KTEEAFALAKEALTVDMKSHICWHVYGILYRQHKNFDEAIKAYKFALKLEPESQQIQRDL
NtNaa15 58 KTEEAFALAKEALTVDMKSHICWHVYGILYRQHKNFDEAIKAYKFALKLEPESQQIQRDL
MmNaa15 61 KKEEAYELVRRGLRNDLKSHVCWHVYGLLQRSDKKYDEAIKCYRNALKWDKDNLQILRDL
RnNaa15 61 KKEEAYELVRRGLRNDLKSHVCWHVYGLLQRSDKKYDEAIKCYRNALKWDKDNLQILRDL
HsNaa15 61 KKEEAYELVRRGLRNDLKSHVCWHVYGLLQRSDKKYDEAIKCYRNALKWDKDNLQILRDL
α8 α9 α10
CtNaa15 118 AVLQIQMRDYAGYVQSRLNMLKARPQIRQNWTALAIAYHLEGNLEKAEHILTTYEKSLTT
CgNaa15 118 AVLQVQMRDYPGYIQSRLVMLKARPQLRQNWTALAIAYHLDGNLQQAENILSTYEKSVTA
MtNaa15 118 AVLQIQMRDYPGYIQSRLLMLKSRPQLRQNWTALAIAYHLDGNLQQAENILTTYEKSVTT
TtNaa15 118 AVLQIQMRDYQGYIQSRLAMLKARPQLRQNWTALAIAYHLDGNLRQAENILTTYEKSVTT
NcNaa15 118 AVLQVQIRDYQGYVQSRFAMLKAKPHLRQNWTALAIAYQLEGSLEQAENILTTYEKSMTN
NtNaa15 118 AVLQVQIRDYQGYVQSRFAMLKAKPHLRQNWTALAIAYQLEGSLEQAENILTTYEKSMTN
MmNaa15 121 SLLQIQMRDLEGYRETRYQLLQLRPAQRASWIGYAIAYHLLEDYEMAAKILEEFRKTQQT
RnNaa15 121 SLLQIQMRDLEGYRETRYQLLQLRPAQRASWIGYAIAYHLLEDYEMAAKILEEFRKTQQT
HsNaa15 121 SLLQIQMRDLEGYRETRYQLLQLRPAQRASWIGYAIAYHLLEDYEMAAKILEEFRKTQQT
α11 α12 α13
CtNaa15 178 PPPKTDLEHSEALLYKNTIIAERGDIERALQHLETDCKHCLDRLAVMELRASYLSKLARK
CgNaa15 178 APLRTDLENSEALLYKNSIIAEMGDFERALEHLEAECKACLDRLAVMELRARYLAELGRK
MtNaa15 178 APLRTDLENSEALLYKNSIIAEMGDFERALEHLETDCKNCLDRLAVMELRARYLTQLGRK
TtNaa15 178 TPLRTDFENSEALLYKNTIIAEMGDIERALEHLETDCKNCLDRLAVMELRARYLAQLGRK
NcNaa15 178 VPLKTDQEHSEALLYKNTIIAERGDIKRALEHLESDCKNCLDRLAVMEMRARYLGQLNRK
NtNaa15 178 VPLKTDQEHSEALLYKNTIIAERGDIKRALEHLESDCKNCLDRLAVMEMRARYLGQLNRK
MmNaa15 181 SPDKVDYEYSELLLYQNQVLREAGLYREALEHLCTYEKQICDKLAVEETKGELLLQLCRL
RnNaa15 181 SPDKVDYEYSELLLYQNQVLREAGLYREALEHLCTYEKQICDKLAVEETKGELLLQLCRL
HsNaa15 181 SPDKVDYEYSELLLYQNQVLREAGLYREALEHLCTYEKQICDKLAVEETKGELLLQLCRL
α14 α15 α16 α17
CtNaa15 238 DEAAKAYRALLDRNPEHMDYYKGLISALDISADDEEAQKAVYDEYAAKYPRSDAAKRLPL
CgNaa15 238 EDAAKAYRALLDRNSEHPDYYKGLVDALGIAADDETALKAVYDD................
MtNaa15 238 EEAAKAYRALLDRNAEHPDYYKGLVDALGTAPDDEVARKAVYDEYAAKNPRCDAAKRLPL
TtNaa15 238 QEAAKAYRALLDRNPEHPEYYKGLINSLGIPAVAEAAQKAVYDEVP..............
NcNaa15 238 EEAAKAWRALLDRNPEHPEYYKGLIEALEIDEKDEAALKAIYDEYAAKYPRSDAAKRLPL
NtNaa15 238 EEAAKAWRALLDRNPEHPEYYKGLIEALEIDEKDEAALKAIYDEYAAKYPRSDAAKRLPL
MmNaa15 241 EDAADVYRGLQERNPENWAYYKGLEKALKPAN..MLERLKIYEEAWTKYPRGLVPRRLPL
RnNaa15 241 EDAADVYRGLQERNPENWAYYKGLEKALKPAN..MLERLKIYEEAWTKFPRGLVPRRLPL
HsNaa15 241 EDAADVYRGLQERNPENWAYYKGLEKALKPAN..MLERLKIYEEAWTKYPRGLVPRRLPL
α18 α19 α20
CtNaa15 298 NFLSGERFRTTAKAYLTLMFDKGVPSTFANLKHLYSDSFKKETLASLAEEYLNEYVNARP
CgNaa15 282 .......FRVAAKAYLTLMFDKGVPSTFANLKHLYSDNFKKETLPILAEEYLEEHRGNSS
MtNaa15 298 DFLSGERFRTAAKAYLTLMFDKGVPSTFANLKHLYSDPFKKDTLPDLAEEYLRERSGTTH
TtNaa15 284 ....GDFFRSTAKEYLTLMFDKGVPSTFANVKHLYSDASKKDILPALAREYLEELRGGSV
NcNaa15 298 NFLTGDSFKAAAKSYLTNMLDRGVPSVFANLKHLYSEVAKKEAILALAEEYLKEHKGSEQ
NtNaa15 298 NFLTGDSFKAAAKSYLTNMLDRGVPSVFANLKHLCSEVAKKEAILALAEEYLKEHKGSEQ
MmNaa15 299 NFLSGEKFKECLDRFLRMNFSKGCPPVFNTLRSLYRDKEKVAIVEELVVGYETSLKSCRL
RnNaa15 299 NFLSGEKFKECLDRFLRMNFSKGCPPVFNTLRSLYRDKEKVAIVEELVVGYETSLKSCRL
HsNaa15 299 NFLSGEKFKECLDKFLRMNFSKGCPPVFNTLRSLYKDKEKVAIIEELVVGYETSLKSCRL

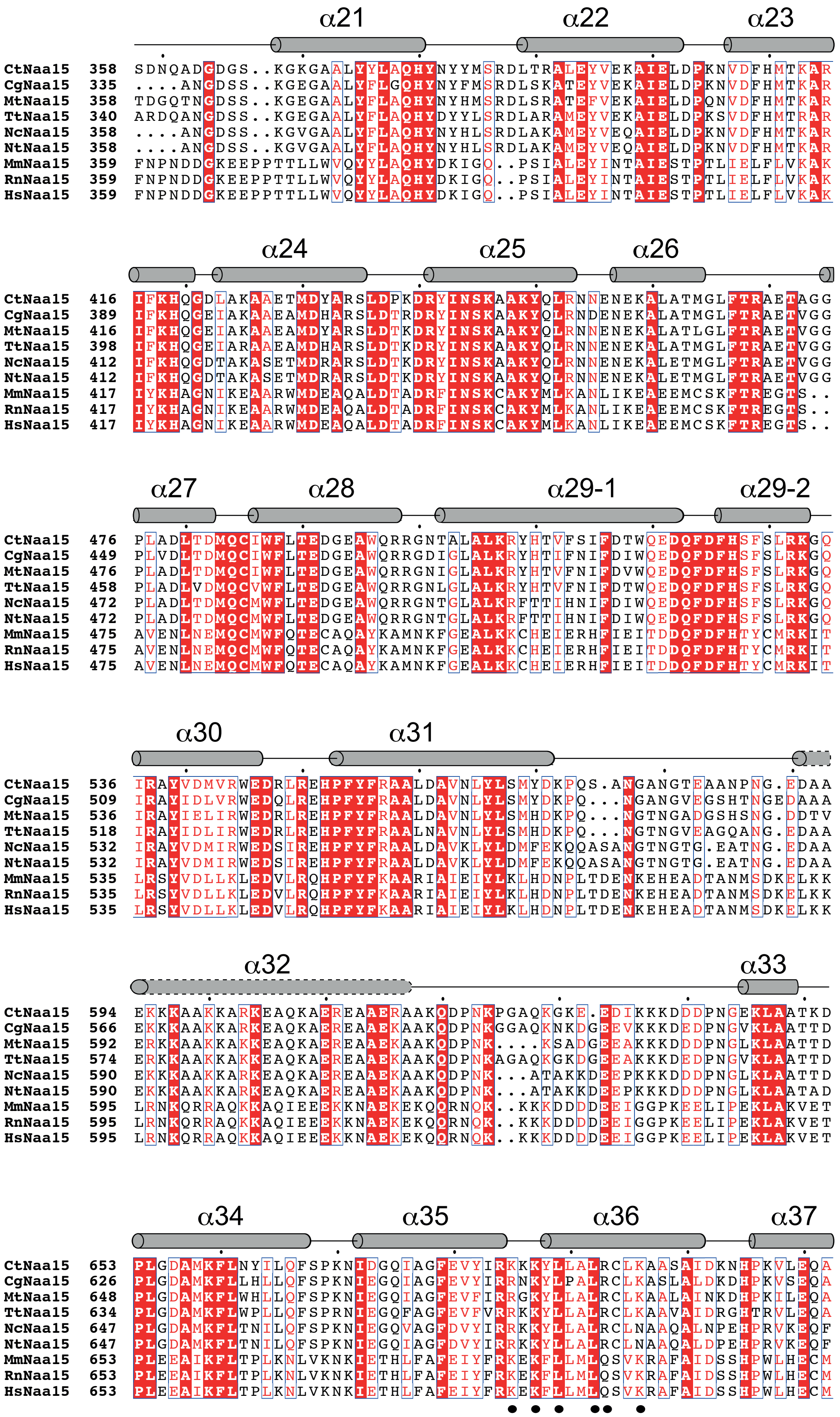
α21
α22
α23
α24
α25
α26
α27
α28
α29-1
α29-2
α30
α31
α32
α33
α34
α35
α36
α37
CtNaa15
CgNaa15
MtNaa15
TtNaa15
NcNaa15
NtNaa15
MmNaa15
RnNaa15
HsNaa15

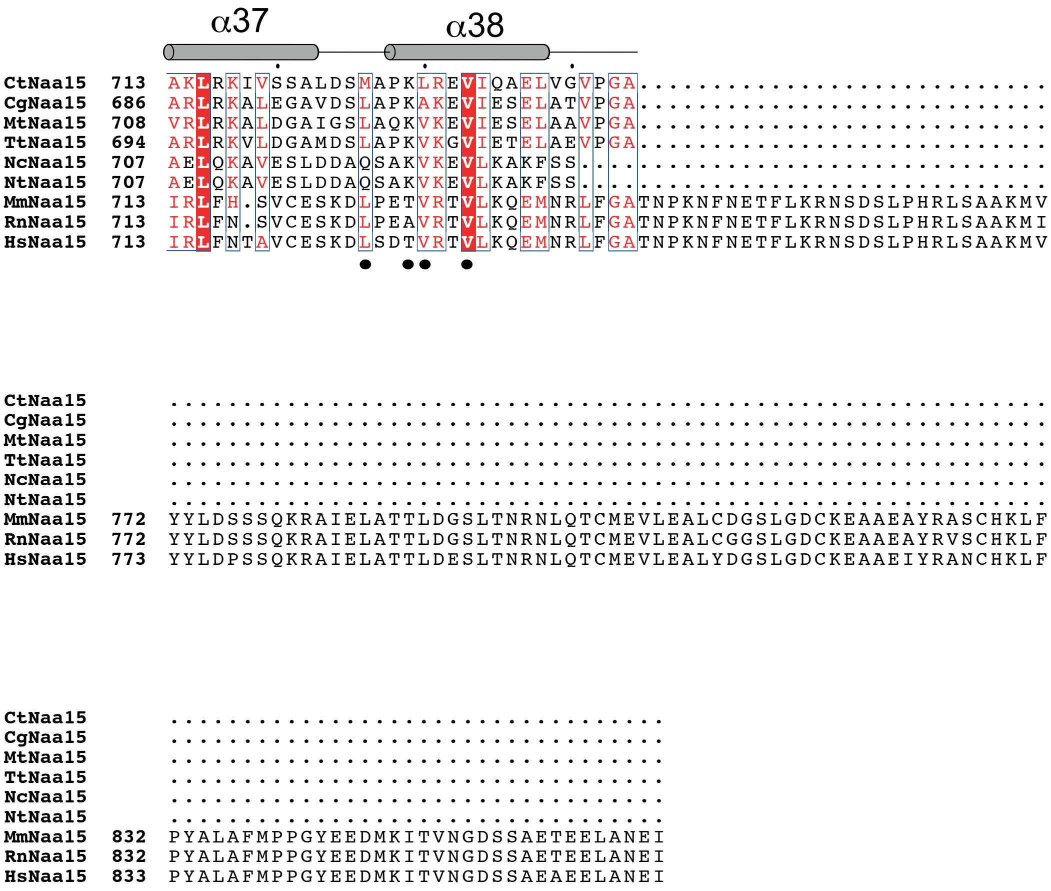

Supplementary Figure 3: Multiple sequence alignment of Naa15 homologs. Highly conserved residues are marked with red boxes. Ct: *Chaetomium thermophilum*, Cg: *Chaetomium globosum,* Mt: *Myceliophthora thermophilia*, Tt: *Thielavia terrestris*, Nc: *Neurospora crassa*, Nt: *Neurospora tetrasperma*, Mm: *Mus musculus*, Rn: *Rattus norvegicus* and Hs: *Homo sapiens*. Multiple sequence alignments were generated using ClustalW2 (ref. 3) and visualized using the ESPript server[4] (http://espript.ibcp.fr). Residues involved in the interaction with HypK are highlighted with a black dot. Secondary structure elements are based on the crystal structure and are represented at the top.

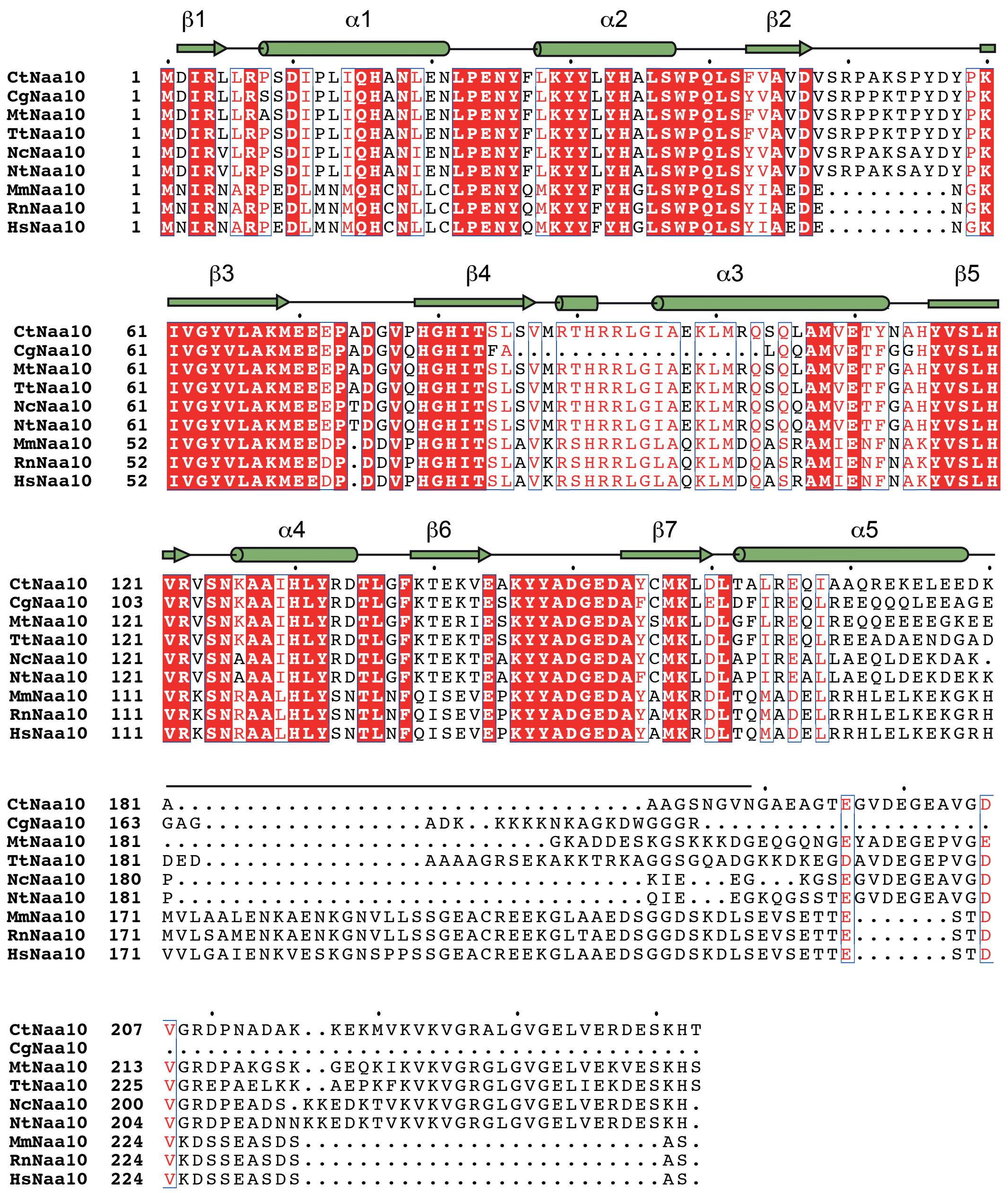

Supplementary Figure 4: Multiple sequence alignment of Naa10 homologs. Highly conserved residues are marked with red boxes. Ct: *Chaetomium thermophilum*, Cg: *Chaetomium globosum,* Mt: *Myceliophthora thermophilia*, Tt: *Thielavia terrestris*, Nc: *Neurospora crassa*, Nt: *Neurospora tetrasperma*, Mm: *Mus musculus*, Rn: *Rattus norvegicus* and Hs: *Homo sapiens*. Multiple sequence alignments were generated using ClustalW2 (ref. 3) and visualized using the ESPript server[4] (http://espript.ibcp.fr). Secondary structure elements are based on the crystal structure (this study) and are represented at the top.

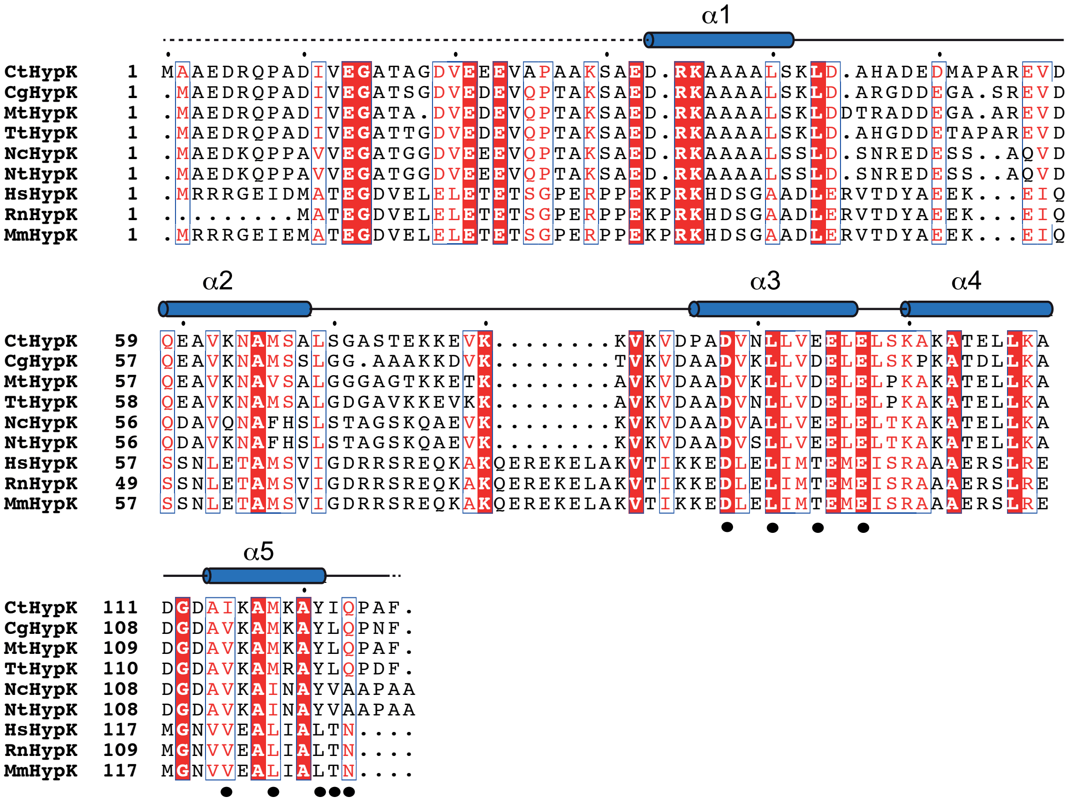

Supplementary Figure 5: Multiple sequence alignment of HypK homologs. Highly conserved residues are marked with red boxes. Ct: *Chaetomium thermophilum*, Cg: *Chaetomium globosum,* Mt: *Myceliophthora thermophilia*, Tt: *Thielavia terrestris*, Nc: *Neurospora crassa*, Nt: *Neurospora tetrasperma*, Hs: *Homo sapiens,* Rn: *Rattus norvegicus* and Mm: *Mus musculus*. Multiple sequence alignments were generated using ClustalW2 (ref. 3) and visualized using the ESPript server[4] (http://espript.ibcp.fr). Residues involved in the interaction with Naa15 are highlighted with a black dot. Secondary structure elements are based on the crystal structure (this study) and are represented at the top.

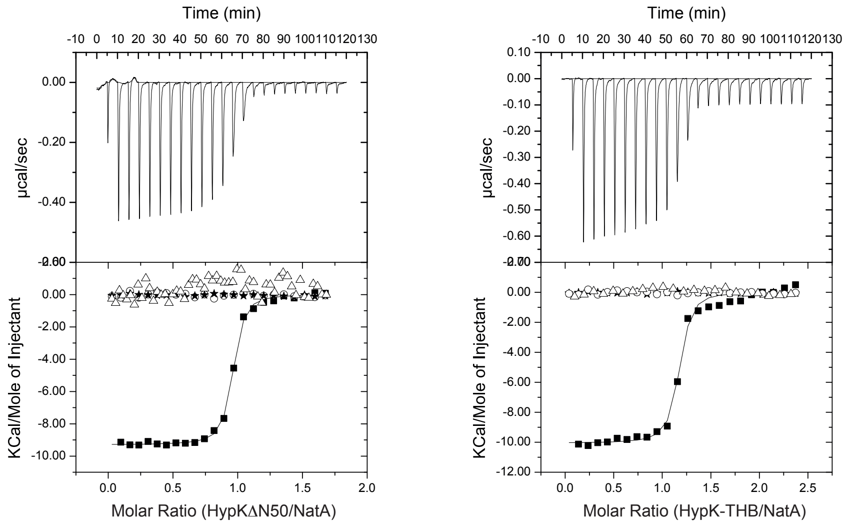

Supplementary Figure 6: Characterization of the interaction between the NatA complex and different HypK-deletion variants by isothermal titration calorimetry. Titration of the NatA complex with HypKΔN50 or HypK-THB (black square). The heats of dilution of the buffer-buffer (black star), buffer-NatA (empty circle) and HypK variants-buffer (empty triangle) are represented.

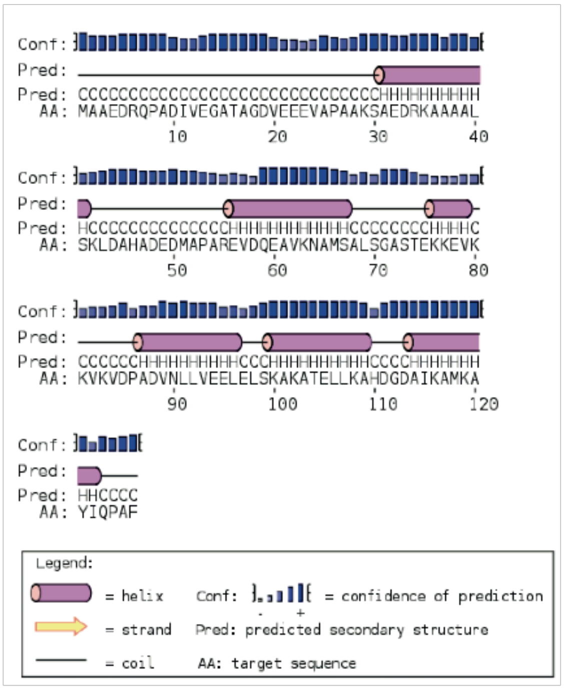

Supplementary Figure 7: Secondary structure prediction of *Ct*HypK. The secondary structure prediction was generated using PSIPRED[5].

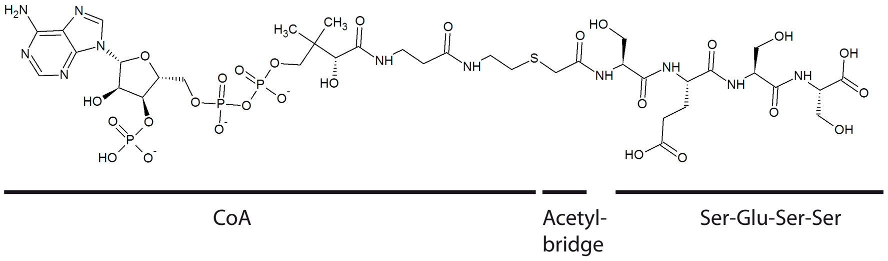

Supplementary Figure 8: Structure of the CoA-SESS bi-substrate[6].

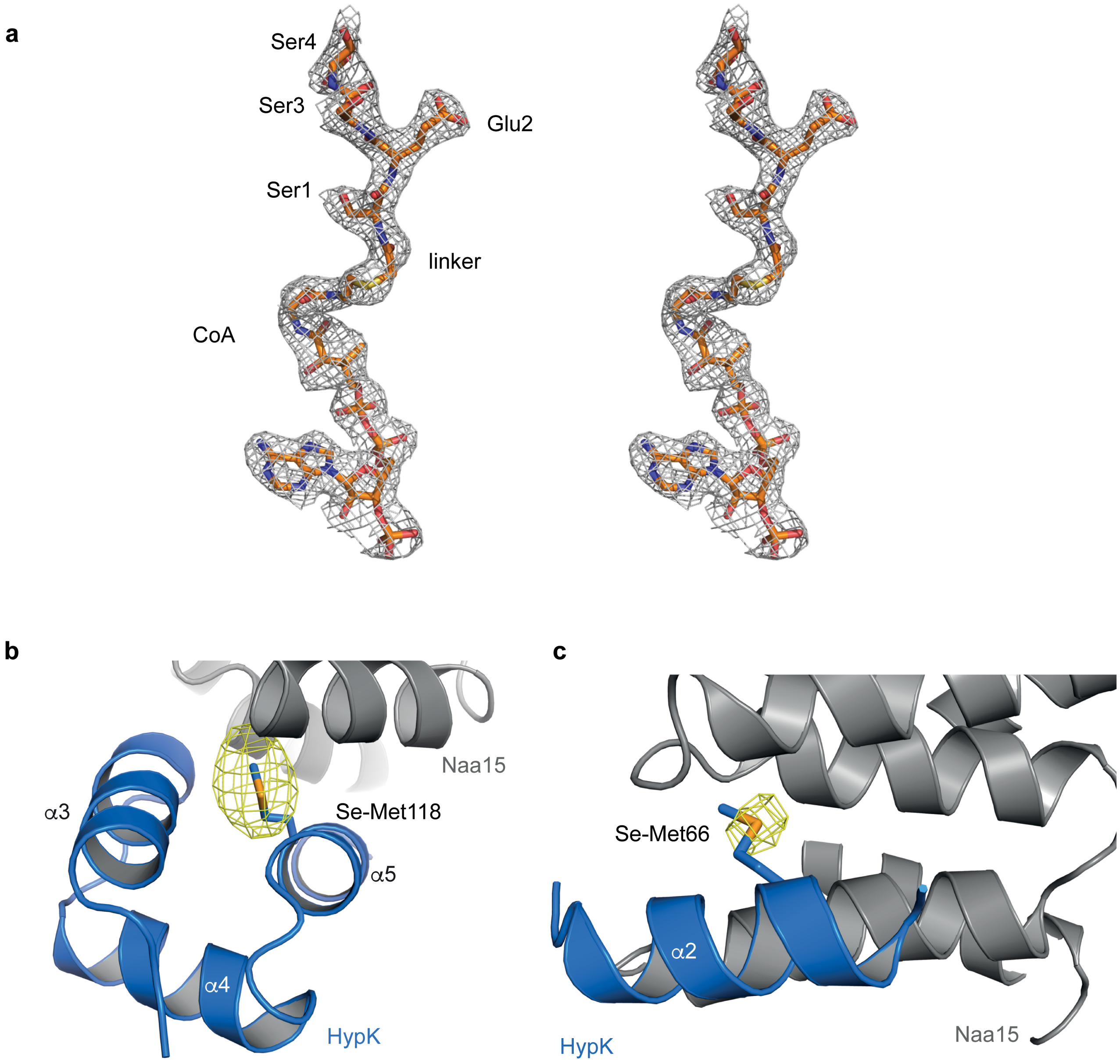

Supplementary Figure 9: Electron density maps of selected regions of HypK. (**a**) Stereo view of the bi-substrate analog CoA-SESS, shown as orange sticks with corresponding electron density at contour level of 1 σ (gray mesh) of a 2mFo-DFc simulated annealing composite omit map. The two CoA-SESS molecules were removed from the model prior to map calculation. The 2mFo-DFc composite omit map was calculated in Phenix[7] using the torsion angle simulated annealing option with an omit fraction of 5% at a time. (**b-c**) Anomalous density map of the seleno-methionine residue Se-Met118 (**b**) and Se-Met66 (**c**) used to assign the sequence is shown at contour level of 5 σ (yellow mesh). HypK, blue; Naa15, gray.

a

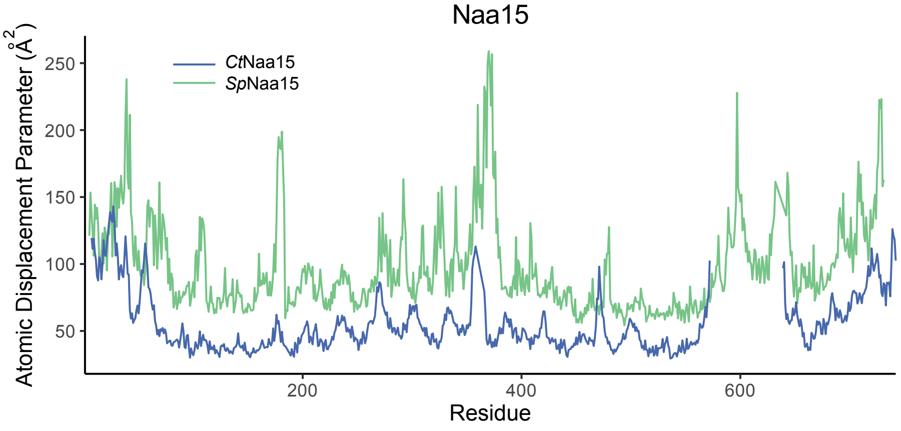

b

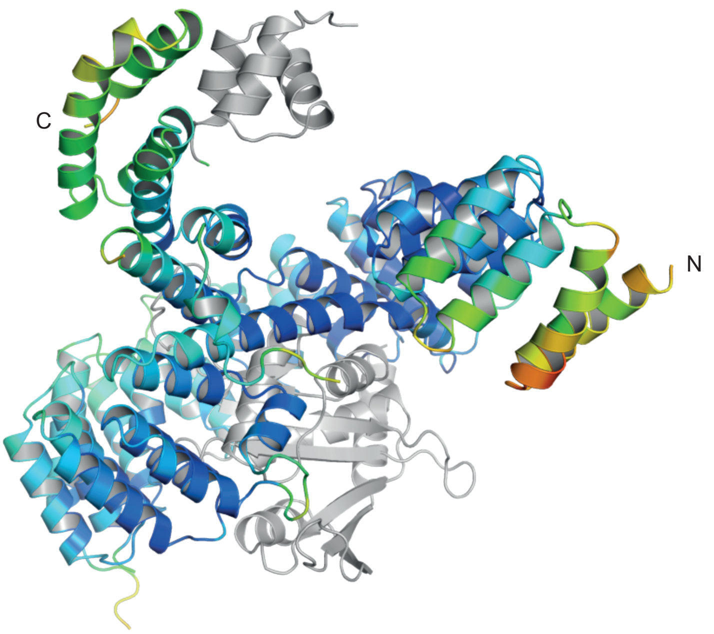

Supplementary Figure 10: B-factor plot for the Naa15 structure. (**a**) Atomic displacement parameter derived dynamics for *Ct*Naa15 (this study) and *Sp*Naa15 (pdb: 4KVM (ref. 8)). The atomic displacement parameters (residue average, calculated with BAVERAGE in CCP4 (ref. 9)) are plotted as a function of the residue number for chain A. (**b**) Structure of HypK-THB - NatA complex. Naa15 is colored according to atomic displacement parameters (residue average), scaled from 20 (blue) to 80 (red) Å^2.

a

b

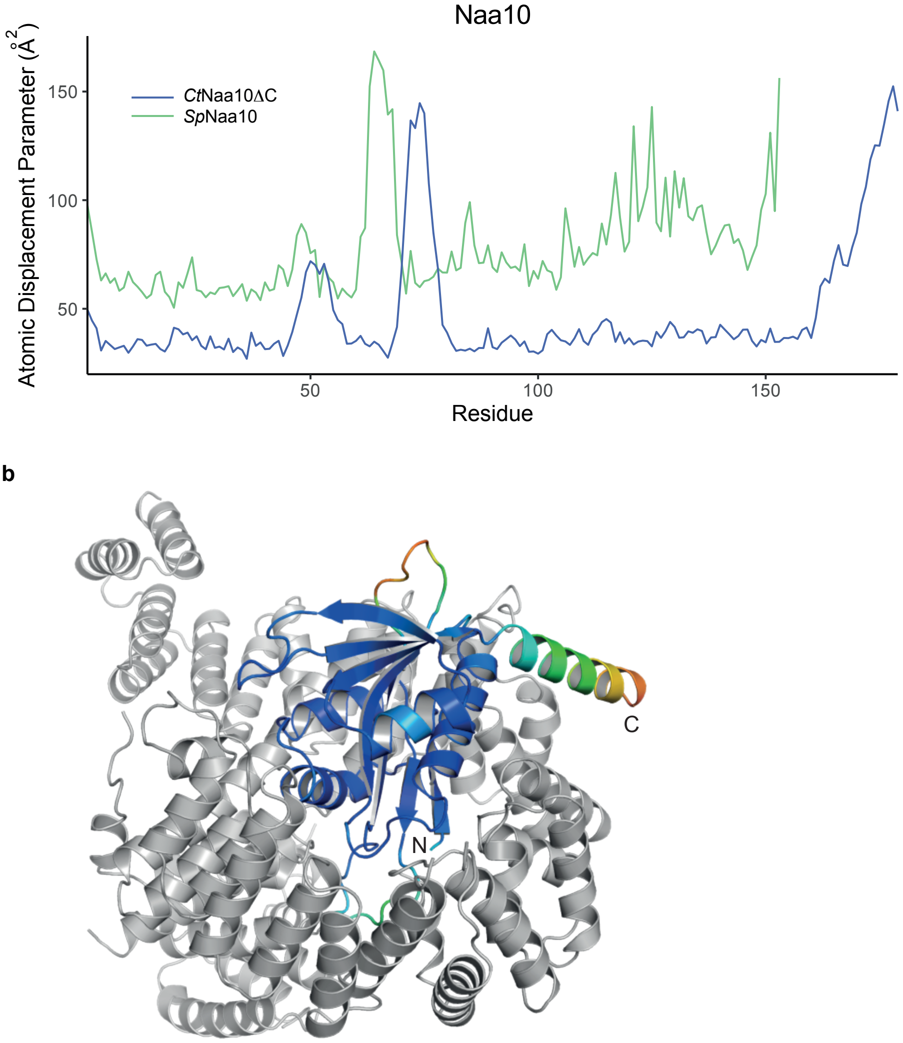

Supplementary Figure 11: B-factor plot for the Naa10 structure. (**a**) Atomic displacement parameter derived dynamics for *Ct*Naa10 (this study) and *Sp*Naa10 (pdb: 4KVM (ref. 8)). The atomic displacement parameters (residue average, calculated with BAVERAGE in CCP4 (ref. 9)) are plotted as a function of the residue number for chain B. (**b**) Structure of HypK-THB - NatA complex. Naa10 is colored according to atomic displacement parameters (residue average), scaled from 20 (blue) to 80 (red) Å^2.

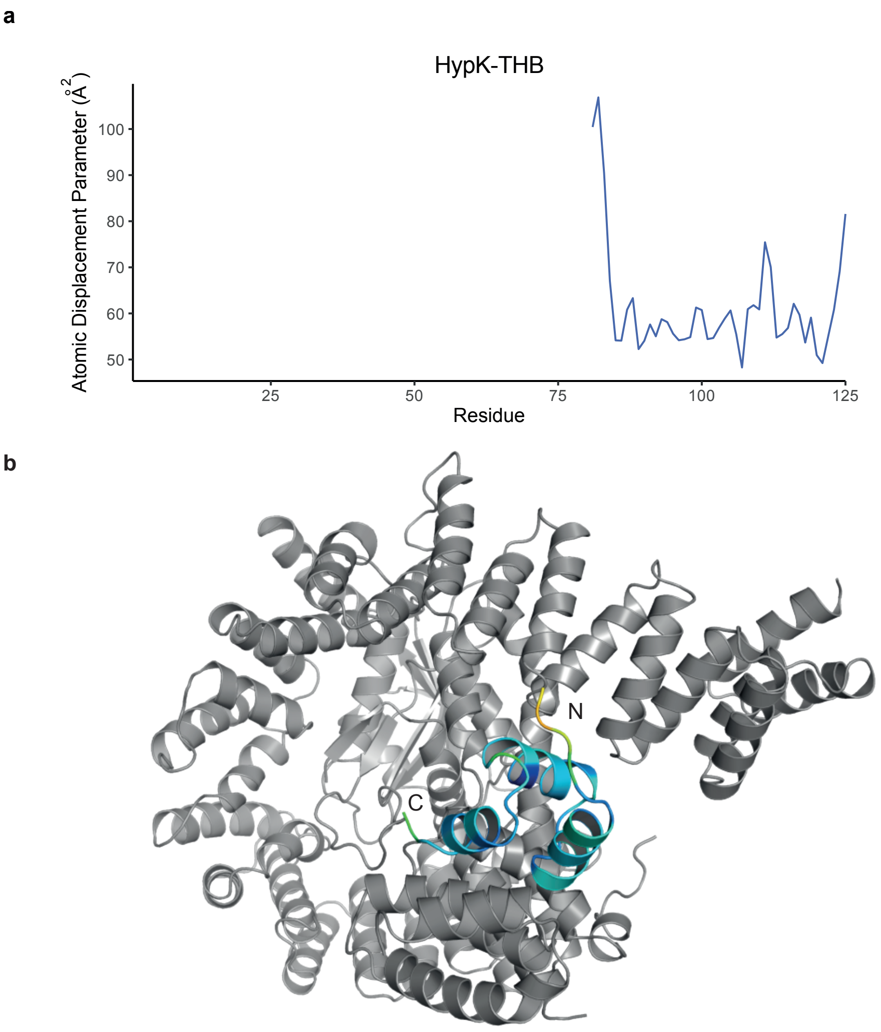

Supplementary Figure 12: B-factor plot for the HypK-THB structure. (**a**) Atomic displacement parameter derived dynamics for HypK-THB. The atomic displacement parameters (residue average, calculated with BAVERAGE in CCP4 (ref. 9)) are plotted as a function of the residue number for chain C. (**b**) Structure of HypK-THB - NatA complex. HypK-THB is colored according to atomic displacement parameters (residue average), scaled from 20 (blue) to 80 (red) Å^2.

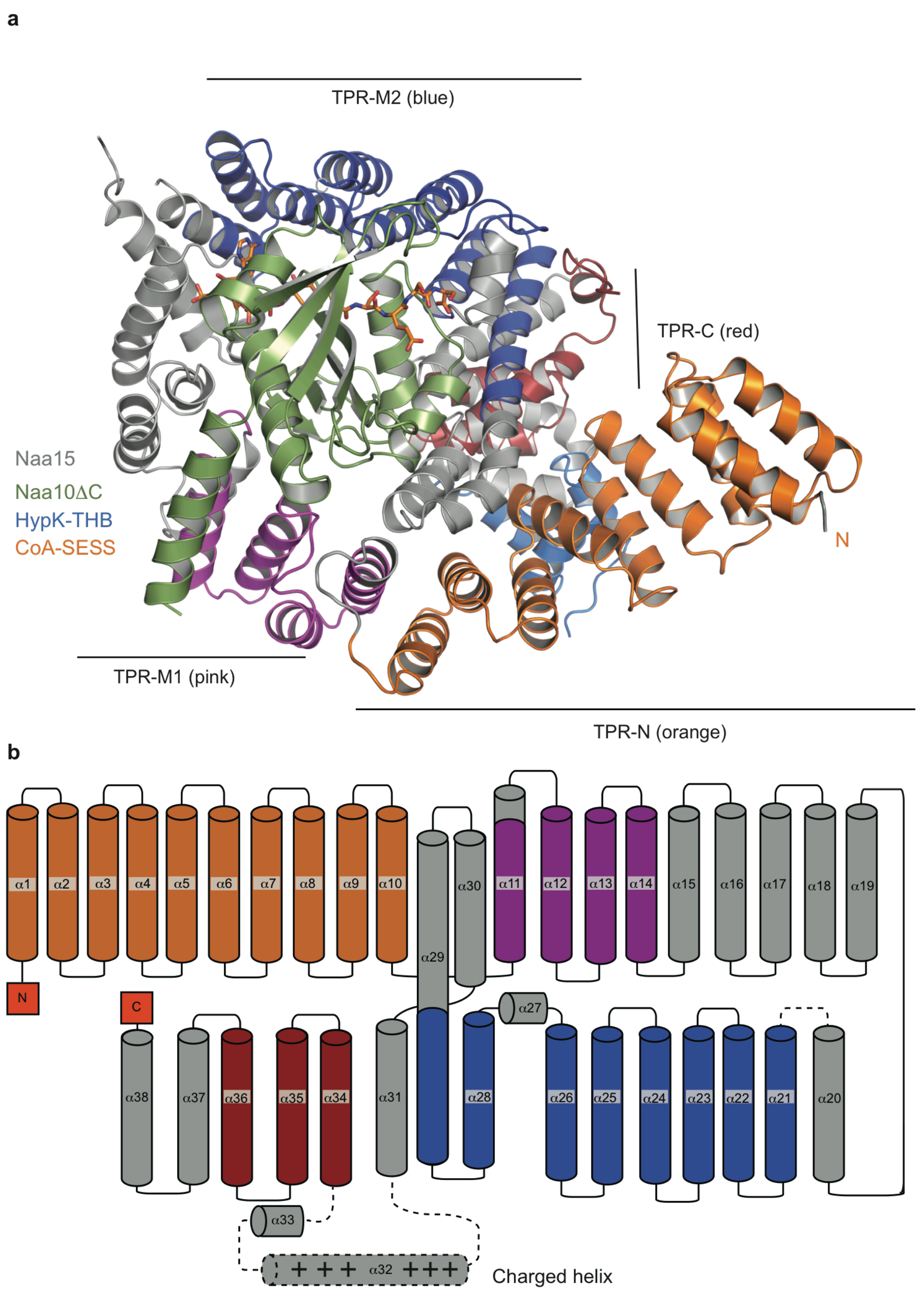

Supplementary Figure 13: Naa15 contains 13 conserved TPR repeats. (**a**) Ribbon representation of the Naa15 (orange, pink, blue, red and gray), Naa10ΔC (green), HypK-THB (light blue) and CoA-SESS (orange sticks) complex structure. The 13 TPR repeats of Naa15 are grouped in four clusters, the N-terminal cluster (TPR-N, orange), the two middle clusters (TPR-M1, pink; TPR-M2, blue) and the C-terminal cluster (TPR-C, red). (**b**) Schematic representation of the topology of Naa15. The TPR-repeats are colored according to (**a**).

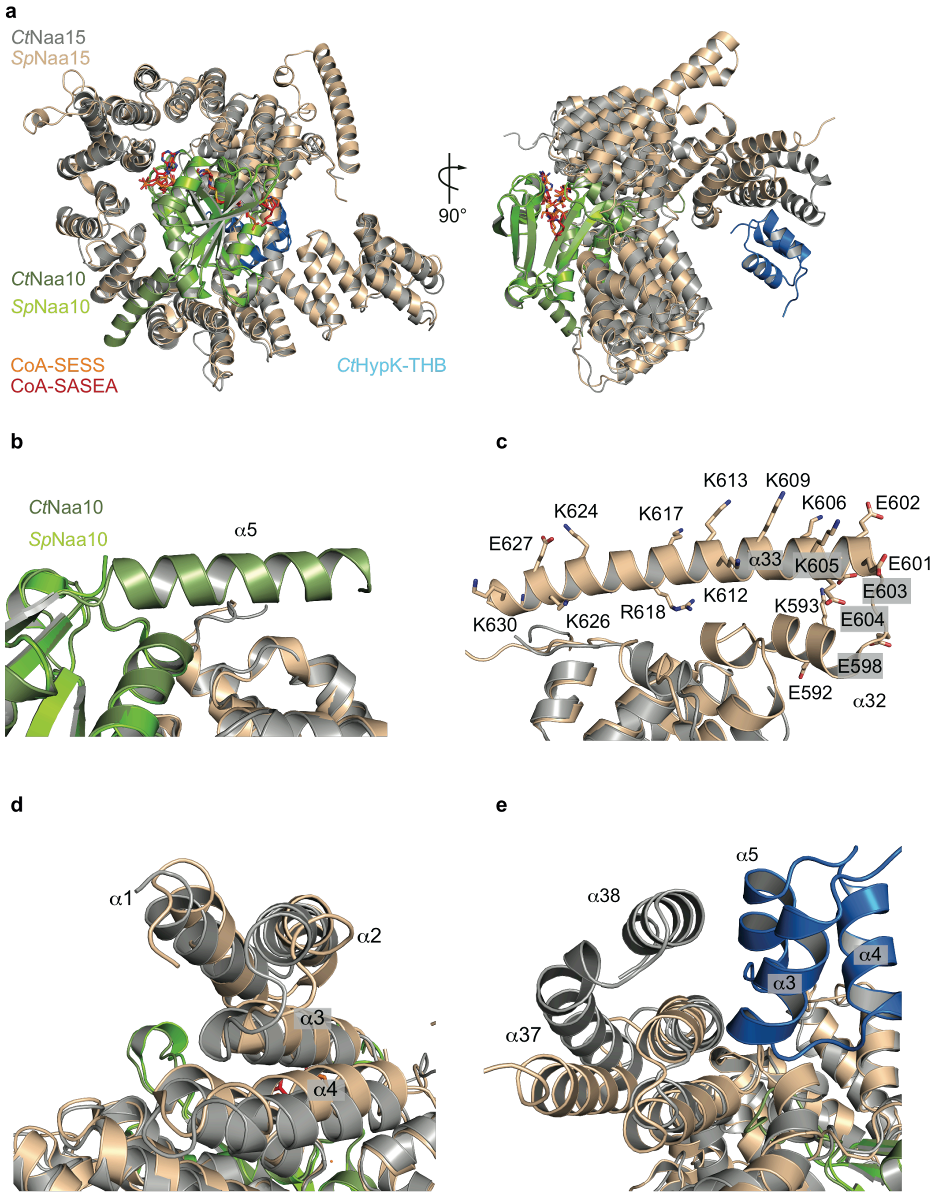

Supplementary Figure 14: Structural comparison between *Ct*NatA and *Sp*NatA. (**a**) Superimposition of the *Ct*NatA - HypK-THB (Naa15 in gray, Naa10 in green, HypK-THB in light blue and CoA-SESS in orange) with the *Sp*NatA homolog (Naa15 in wheat, Naa10 in mint and CoA-SASEA in red, pdb: 4KVM (ref. 8)). (**b**) Close-up view on the helix α5 of *Ct*NatA structure, omitted in the construct used for *Sp*NatA (pdb: 4KVM (ref. 8)). **(c)** Close up view on the helices α32 and α33. The highly mobile helices α32 and α33 are not resolved in the *Ct*NatA structure, but in the *Sp*NatA structure. The positions of the charged residues are labeled. (**d**) Close up view on the N-terminal rearrangement of Naa15. Helix α1- α4 of *Ct*Naa15 are rearranged in comparison with the structure of *Sp*Naa15. (**e**) Close up view on the C-terminal rearrangement of Naa15. The additional helix α38 of *Ct*Naa15 is visible.

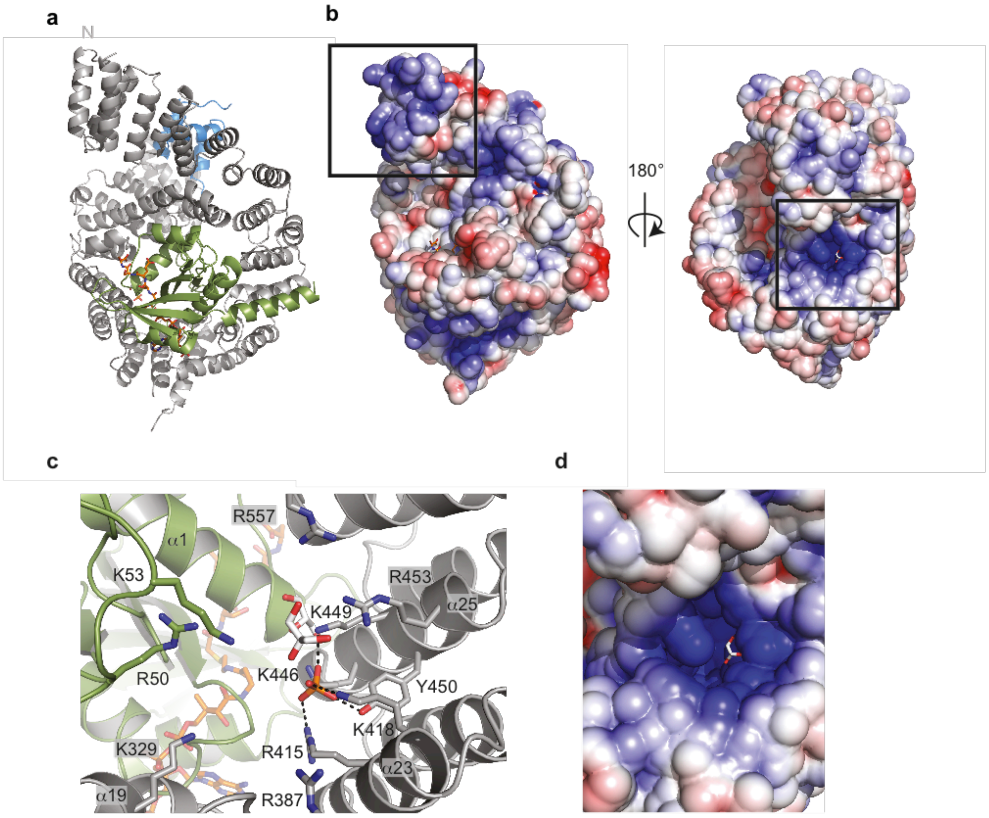

Supplementary Figure 15: Surface charge distribution of the HypK-THB - Naa15-Naa10ΔC complex. (**a**) Cartoon representation of the heterotrimeric complex. Naa15 in gray, Naa10ΔC in green, HypK-THB in blue and CoA-SESS (orange sticks). (**b**) Electrostatic surface potential of the HypK-THB - Naa15-Naa10ΔC complex. The highly charged patch at the N-terminus of Naa15 is highlighted with a black box. The two glycerol molecules (white sticks) and phosphate ion (orange sticks) are shown. The surface charge spans from -5 kT/e (deep red) to +5 kT/e (deep blue). (**c**) Close up view of the charged cavity formed by lysine and arginine residues of Naa15 and Naa10. Residues forming the cavity are labeled and the coordination of the phosphate ion is shown as black lines. (**d**) Close up view of the charged cavity as electrostatic surface potential.

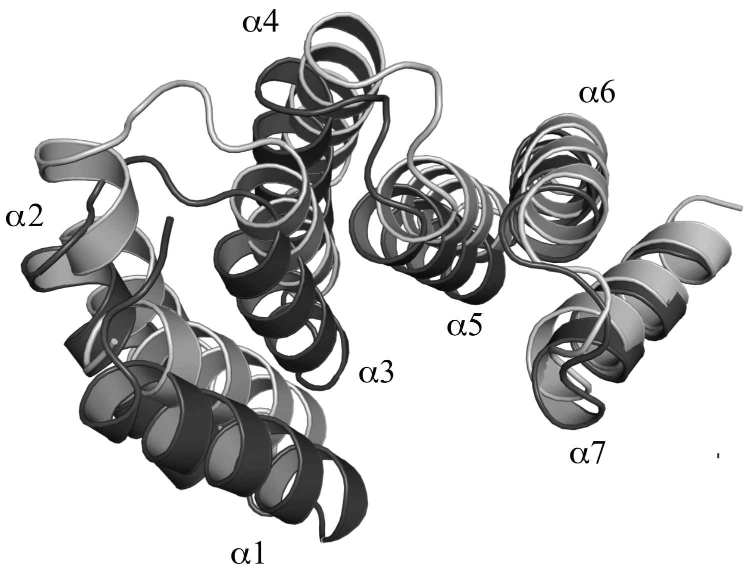

Supplementary Figure 16: Superimposition of Naa15 structures. Cartoon representation of the Naa15 N-terminal region (helix α1-7) of the HypK-THB - NatA complex (light gray) superimposed on HypK as part of the full-length HypK - NatA complex (dark gray).

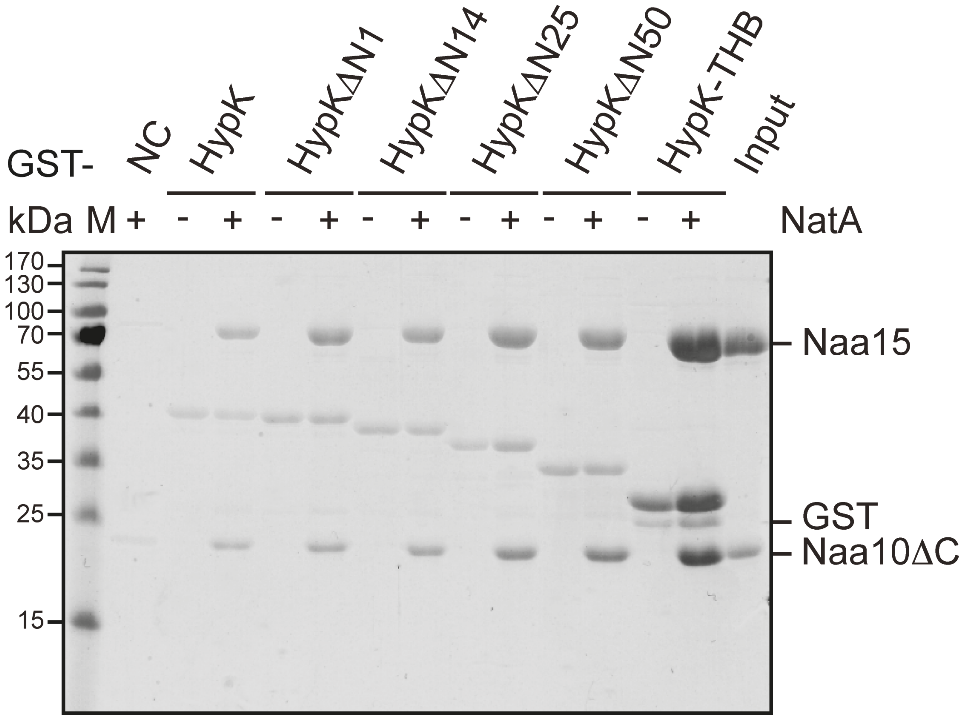

Supplementary Figure 17: Uncropped image of Fig. 1a.

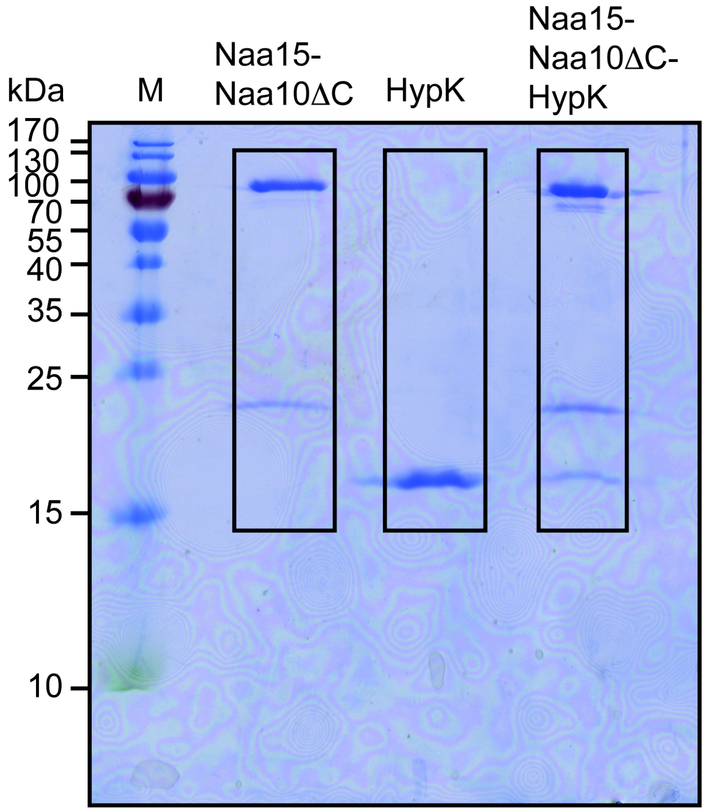

Supplementary Figure 18: Uncropped image of Supplementary Fig. 2.

Supplementary References

1. Biegert, A. *et al.* The MPI Bioinformatics Toolkit for protein sequence analysis. *Nucleic Acids Res.* **34**, W335-339 (2006).
2. Krissinel, E. & Henrick, K. Inference of macromolecular assemblies from crystalline state. *J. Mol. Biol.* **372**, 774-797 (2007).
3. Sievers, F. *et al.* Fast, scalable generation of high-quality protein multiple sequence alignments using Clustal Omega. *Mol. Syst. Biol.* **7**, 539 (2011).
4. Robert, X. & Gouet, P. Deciphering key features in protein structures with the new ENDscript server. *Nucleic Acids Res.* **42**, W320-324 (2014).
5. Buchan, D.W. *et al.* Scalable web services for the PSIPRED Protein Analysis Workbench. *Nucleic Acids Res.* **41**, W349-357 (2013).
6. Foyn, H. *et al.* Design, Synthesis, and Kinetic Characterization of Protein N-Terminal Acetyltransferase Inhibitors. *ACS Chem. Biol.* (2013).
7. Adams, P.D. et al. PHENIX: a comprehensive Python-based system for macromolecular structure solution. *Acta Crystallogr. D Biol. Crystallogr.* **66**, 213-221 (2010).
8. Liszczak, G. *et al.* Molecular basis for N-terminal acetylation by the heterodimeric NatA complex. *Nat. Struct. Mol. Biol.* **20**, 1098-1105 (2013).
9. Winn, M.D. *et al.* Overview of the CCP4 suite and current developments. *Acta Crystallogr. D Biol. Crystallogr.* **67**, 235-242 (2011).
